# Hybridization Capture Reveals Evolution and Conservation across the Entire Koala Retrovirus Genome

**DOI:** 10.1371/journal.pone.0095633

**Published:** 2014-04-21

**Authors:** Kyriakos Tsangaras, Matthew C. Siracusa, Nikolas Nikolaidis, Yasuko Ishida, Pin Cui, Hanna Vielgrader, Kristofer M. Helgen, Alfred L. Roca, Alex D. Greenwood

**Affiliations:** 1 Department of Wildlife Diseases, Leibniz Institute for Zoo and Wildlife Research, Berlin, Germany; 2 Department of Biological Science and Center for Applied Biotechnology Studies, California State University, Fullerton, California, United States of America; 3 Department of Animal Sciences, University of Illinois at Urbana-Champaign, Urbana, Illinois, United States of America; 4 Zoo Vienna, Vienna, Austria; 5 National Museum of Natural History, Smithsonian Institution, Washington, DC, United States of America; Institut Pasteur, France

## Abstract

The koala retrovirus (KoRV) is the only retrovirus known to be in the midst of invading the germ line of its host species. Hybridization capture and next generation sequencing were used on modern and museum DNA samples of koala (*Phascolarctos cinereus*) to examine ca. 130 years of evolution across the full KoRV genome. Overall, the entire proviral genome appeared to be conserved across time in sequence, protein structure and transcriptional binding sites. A total of 138 polymorphisms were detected, of which 72 were found in more than one individual. At every polymorphic site in the museum koalas, one of the character states matched that of modern KoRV. Among non-synonymous polymorphisms, radical substitutions involving large physiochemical differences between amino acids were elevated in *env,* potentially reflecting anti-viral immune pressure or avoidance of receptor interference. Polymorphisms were not detected within two functional regions believed to affect infectivity. Host sequences flanking proviral integration sites were also captured; with few proviral loci shared among koalas. Recently described variants of KoRV, designated KoRV-B and KoRV-J, were not detected in museum samples, suggesting that these variants may be of recent origin.

## Introduction

Endogenous retrovirus-like elements (ERVs) are common in the genomes of vertebrates, comprising 8% of the human genome [1]. ERVs derive from retroviruses that invaded the germ line of ancestral host organisms, becoming permanent genomic elements in the host lineage. Although most ERVs have adapted to become non-pathogenic and non-functional in their host, a role in human health and disease has been established for some ERVs [2], [3]. One ERV in the human germ line has been co-opted as a functional gene, *syncytin*, which is critical for normal development of the human placenta [4]. Recently, another human ERV has been found to play a critical role in the progression of Hodgkin’s lymphoma [5]. Despite their biomedical importance, the process by which ERVs invade their host germ lines has been difficult to study, given that almost all known ERVs are many thousands or millions of years old.

The only retrovirus known to be in the midst of transitioning from an exogenous to an endogenous form is the koala retrovirus (KoRV). KoRV is currently invading the germ line of its host species, the koala (*Phascolarctos cinereus*), but is not found in the genomes of all koalas [3], [6]–[8]. KoRV is ubiquitous among northern Australian koalas, but is less common in southern Australian mainland and island populations [8]–[10]. PCR and sequencing of KoRV *env* genes in museum specimens of koalas from the late 1800s revealed that KoRV was already ubiquitous among northern Australian koalas at that time [6]. While *env* has been examined in historical samples, little is known about the historical variability or stability of the rest of the KoRV genome or changes in integration site diversity over time.

Two protein motifs, one in Gag and another in Env, have been associated with reduced infectivity of KoRV relative to the closely related gibbon ape leukemia virus (GALV). A CETTG motif in GALV Env is highly conserved across gammaretroviruses, while SRLPIY in GALV Gag is associated with promoting viral release [3]. Both protein motifs differ between KoRV and GALV, and these differences are believed to lower the relative infectivity of KoRV [3]. In historical samples of koalas, both motifs matched that of modern koalas, with no differences or polymorphisms detected in koala samples going back to the late 1800s [6]. The reduced virulence of KoRV relative to GALV, and the lack of historical polymorphisms, has led to a hypothesis that the changes to these two protein domains may have both preceded and enabled the invasion of the koala germ line by KoRV.

Several laboratories have recently reported novel variants of KoRV [11], [12]. One variant has been designated KoRV-B, with the originally identified KoRV labeled KoRV-A [12]. KoRV-B has greater virulence than KoRV-A, and has been isolated only from a subset of the koalas housed at the Los Angeles Zoo, and not from wild koalas. The KoRV-B long terminal repeat (LTR) U3 region includes 4 repeats of a core enhancer element, whereas KoRV-A has only one. The KoRV-B Env also has a different receptor-binding domain [12]. KoRV-B has the CETTG motif that is present in other infectious gammaretroviruses, but that has the sequence CETAG in KoRV-A. While KoRV-A uses the sodium dependent phosphate transporter membrane protein (PiT-1 or SLC20A1) as a receptor for viral entry, KoRV-B uses the thiamine transporter protein 1 (THTR1 or SLC19A2) [12]. Another recently identified variant, designated KoRV-J, also utilizes the THTR1 receptor for viral entry although KoRV-J does not have the CETTG motif of KoRV-B [11]. KoRV-J has been detected in zoo koalas [11]. Both KoRV-J and KoRV-B may be recently arisen variants, differing from KoRV-A in the LTR and *env* sequences, although they have not been examined in historical samples.

KoRV variants such as KoRV-B show differences in regions beyond *env* and thus, it would be of interest to characterize polymorphisms, not just for *env*, but also across *gag*, *pol,* LTRs, and the koala genomic sequences flanking KoRV proviral loci. However, PCR based methods are labor intensive and often unsuccessful when applied to historical samples. To examine KoRV evolution, we here applied a hybridization capture method to modern and ancient koala DNA, including multiple koala specimens in a single next generation sequencing run, in order to capture DNA sequences spanning the full length of the KoRV proviral genome. Recently developed solution hybridization capture methods allow for the specific enrichment of target sequences from genomic libraries, using PCR amplicons as “bait” to which target DNA hybridizes [13], [14]. Even when the target sequences is divergent, both long (200–500 nt) and short (<30 nucleotide) DNA fragments can be captured and sequenced efficiently [15], allowing use of the method with both modern and ancient DNA. This enabled us to characterize polymorphisms across the entire KoRV genome and koala genomic sequences flanking KoRV proviral loci. Polymorphisms were analyzed, and used to model potential changes to protein structure, or to identify potential changes to transcription factor binding sites in the LTRs. The flanking sequence data was used to identify integration sites common to more than one koala, identifying endogenous loci. Hybridization capture also allowed us to investigate whether KoRV-B, KoRV-J, and other recently described variants [11], [12] were present in a modern deep sequenced koala, or in historical samples.

## Materials and Methods

### Koala Samples and DNA Extraction

Archival and modern samples are described in Table 1. All archival samples were extracted in a dedicated ancient DNA laboratory in the Department of Wildlife Diseases of the Leibniz Institute for Zoo and Wildlife Research under plexiglass UV hoods dedicated to DNA extraction. The ancient DNA laboratory was never used for molecular or genetic work on modern samples, and followed procedures designed to minimize the possibility of contamination, such as wearing protective clothing during extractions to avoid contamination from the researchers. Each extraction involved approximately 250 mg of dried skin, and used the Geneclean Ancient DNA extraction kit from MP Biomedicals, USA, following the manufacturer’s protocol. Mock extractions were performed for each set of museum specimens as controls for potential contamination during the extraction process. Each DNA extract was further purified using Qiaquick spin columns (Qiagen) as described previously [16]. DNA extraction from a blood sample of modern koala Pci-SN265 (zoo koalas in North America and Europe are included in the North American regional studbook, and are here designated by studbook number, “SN”) was performed in a separate laboratory in a different floor of the Leibniz Institute for Zoo and Wildlife Research. This extraction was performed using the Qiagen DNeasy Blood & Tissue Kit following the manufacturer’s protocol. The extracted DNA was then fragmented using a Covaris-S220 to generate 150 bp fragments.

**Table 1:** Koala sample information.

Sample number	North/South Australia	Sample type	Sample provider/wild locality	Collection date	Full KoRV	Prior *env*	Flanking sequence	Used for bait
Pci-QM-J6480	North	Wild-museum	Queensland Museum	1938	+		+	
Pci-MCZ-12454	North	Wild-museum	Museum of Comparative Zoology	1904	+	+	+	
Pci-MCZ 8574	North	Wild-museum	Museum of Comparative Zoology	1904	+		+	
Pci-582119	North	Wild-museum	Stockholm Museum	1911	+	+	+	
Pci-c2831	North	Wild-museum	Museum of Victoria	1923	−			
Pci-c2832	North	Wild-museum	Museum of Victoria	1923	−			
Pci-um3435	North	Wild-museum	Bohusläns Museum	1891	+	+	+	
Pci-AM-M1461	South	Wild-museum	Australian Museum/NSW	1883	−			
Pci-AM-B4593	South	Wild-museum	Australian Museum/NSW	1884	−			
Pci-maex1738	North	Wild-museum	Goteborg Museum	1870–1891	+	+	+	
Pci-SN265	North	Zoo-modern	Schönbrunn Zoo Vienna (Mirra-Li)	2012	+		+	
Pci-SN345	North	Zoo-modern	San Diego Zoo (USA)	2010			+	
Pci-SN404	North	Zoo-modern	San Diego Zoo (USA)	2010			+	+
Pci-SN248	North	Zoo-modern	San Diego Zoo (USA)	2010			+	
Pci-142	South	Wild-modern	NCI/Stony Rises	1990s				+
Pci-157	South	Wild-modern	NCI/Stony Rises	1990s			+	+
Pci-106	South	Wild-modern	NCI/Brisbane Ranges	1990s			+	+
Pci-182	South	Wild-modern	NCI/Kangaroo Island	1990s			+	

Koala sample numbers were based on studbook numbers (“SN”) for zoos, and specimen numbers for each museum. Wild modern samples were from the National Cancer Institute (NCI), and have NCI codes. North Australian samples are from Queensland; NSW is New South Wales. Collection dates for archival samples were confirmed by K.H. from museum records; date ranges listed are as exact as possible given museum records. Plus sign indicates successful attempt; minus sign indicates attempted unsuccessfully; blank indicates not attempted. Prior *env* sequences refer to those derived from PCR and reported in [6]. Flanking sequences for modern koala samples are from Ishida et al. (submitted).

Blood samples of San Diego Zoo koalas were collected during routine physical exams and genomic DNA was isolated from buffy coat using the Qiagen DNeasy Blood & Tissue Kit following the manufacturer’s protocol. DNA from blood samples of wild koalas had been extracted using a phenol-chloroform method. These samples were used to generate baits.

### Ethics Statement

All experiments involving koala tissues were approved by the Internal Ethics Committee of the Leibniz Institute for Zoo and Wildlife Research, approval number 01-01-2013. Work involving other modern koala samples was conducted at the University of Illinois at Urbana-Champaign (UIUC), under IACUC approval number 12040.

### Polymerase Chain Reaction

All museum specimen were initially screened for a KoRV *pol* fragment by PCR (Table 1) performed in a volume of 34 µl using 5.5 µl of extract, 10 nm of primers, 0.5 U Platinum HiFi supermix (Invitrogen), 1µl of bovine serum albumin (Fermentas), and 1 µl of primers P1aF ‘5-TTGGAGGAGGAATACCGATTACAC-3′ with P1aR ‘5-GCCAGTCCCATACCTGCCTT-3′ [8]. Cycling conditions were: 94°C for 4 min; 60 cycles at 94°C for 30 s, 55°C for 30 s, 72°C for 30 s; and 72°C for 10 min, with the samples then held at 4°C [17]. The high cycle number (60) PCR was only used for screening museum koala samples for the presence of KoRV and not for polymorphism analyses. The modern sample was screened by PCR amplification performed in a volume of 34 µl using 1 µl (26.7 ng/µl) of extract, 10 nM of each primer, 0.5 U of Platinum HiFi supermix (Invitrogen). Cycling condition were: 94°C for 4 min; 35 cycles at 94°C for 30 s, 55°C for 30 s, 72°C for 30 s; and 72°C for 5 min, with the samples then held at 4°C. PCR products were visualized on a 3% gel. All gels used GelRed nucleic acid gel stain by Biotium. PCR products were purified using the NucleoSpin Gel and PCR Clean up kit (Macharey-Nagel). PCR products were commercially sequenced by the Sanger method using the forward and reverse PCR primers (StarSeq, Germany). Primers used in this study are listed in Table S1. The Sanger sequences were not included in the hybridization capture alignments but were only used to establish the presence of KoRV in museum and modern samples.

### Illumina Library Preparation

Aliquots from each DNA extract were used in generating Illumina libraries. Archival extract libraries were generated in the ancient DNA facility in a library-dedicated plexiglass PCR UV hood, while the modern koala library was generated in a modern DNA laboratory in a different part of the Institute. Libraries were generated as described in Mayer et al. [18]. Each library contained a unique index adapter to allow for subsequent discrimination among samples after the sequencing of pooled libraries. A negative control extraction library was also prepared and indexed separately to monitor any contamination introduced during the experiment. Indexes were added by PCR using Amplitaq Gold DNA polymerase (Applied Biosystems [ABI]) in 100 µl reactions. Cycling condition were: 94°C for 5 min; 10 cycles at 94°C for 30 s, 55°C for 30 s, 72°C for 30 s; and 72°C for 5 min; the samples were then held at 4°C. After indexing, the samples would effectively be at little or no risk from cross contamination either from the other libraries or from laboratory DNA. Quantitative PCR (qPCR) was performed after index PCR with a standard that was developed using 100 bp PCR product with Illumina primer binding sites ligated at the 5′ and 3′ ends as described in Mayer et al. [18]. The qPCR standard curve was obtained using a series dilution of the standard. The assay was performed in a Stratagene MxPro 3000p qPCR system using Brilliant III Ultra-Fast SyBr Green qPCR master mix (Agilent) with Illumina bridge primers P5 and P7 [18] to determine the number of molecules in each sample. Additional amplification was followed using Herculase II DNA polymerase (Agilent) with P5 and P7 Illumina library outer primers with the same cycling conditions. DNA products were purified using Minelute columns (Qiagen) after each amplification step. Final quantification was performed on an Agilent 2200 tape station D1K tape.

### Primer Design and Preparation of Baits

PCR products used as “bait” for capturing sequences from the Illumina libraries were generated at the University of Illinois to limit the amount of koala and KoRV amplicons present in the laboratories in Berlin. DNA of one northern koala, Pci-SN404 (see above) and three southern koalas (PCI-157 and PCI-142 from the Stony Rises and PCI-106 from the Brisbane Ranges of southern Australia) were used in preparing the bait. Primers were newly designed to cover the complete KoRV genome outside the *envelope* region. For the *envelope* region, previously designed primers were used [6] but with primer combinations that would yield amplicon sizes of approximately 500 bp. For the other KoRV regions, novel primers based on the published KoRV sequence (GenBank: AF151794) [7] were designed using Primer3 (http://fokker.wi.mit.edu/primer3/input.htm) [19] to yield amplicons of approximately 500 bp. The KoRV genome was amplified in thirty-eight 500 bp overlapping products using the primers shown in Table S1. The PCR mix consisted of 1 X PCR Buffer II (ABI), 1.5 mM MgCl_2_ (ABI), 0.4 µM of final concentration of each primer, 200 µM of each dNTP (ABI), with 0.04 units/µl final concentration of AmpliTaq Gold DNA Polymerase (ABI). The PCR algorithm consisted of an initial 95°C for 10 min; with cycles of 15 sec at 95°C; followed by 30 sec at 60°C, 58°C, 56°C, 54°C, 52°C (2 cycles at each temperature) or 50°C (last 30 cycles); and 1 min at 72°C; with a final extension of 7 min at 72°C. An aliquot of each PCR product was visualized on a 1% agarose gel with ethidium bromide. PCR products were enzyme-purified [20] and Sanger-sequenced to verify that the target region had been amplified. The PCR products were purified using Qiaquick columns (Qiagen) and then quantified using a NanoDrop ND-1000 (Thermo-Scientific). KoRV amplicons were then blunt-ended, ligated to a biotin adapter, and immobilized on streptavidin magnetic beads in equimolar amounts of 1.3 µg as described previously [14].

### Hybridization Capture

Mixtures of blocking agent, blocking oligos, and indexed koala libraries were heated to 95°C to separate the DNA strands [14]. One aliquot from each index library was mixed with streptavidin beads bound with biotinylated KoRV PCR products. Samples were incubated for 48 hours at 65°C under rotation in a Labnet mini incubator. After 48 hours the beads were washed and the hybridized libraries eluted by heating. The DNA concentration was measured by quantitative PCR (qPCR), and the eluted libraries were further amplified accordingly using P5 and P7 Illumina outer primers. The products were then pooled at equimolar concentrations for paired-end sequencing on an Illumina MiSeq platform at the National High-Throughput DNA Sequencing Center, University of Copenhagen.

### Sequence Assembly, Identification of Polymorphisms and Integration Site Analysis

**Figure 1:**
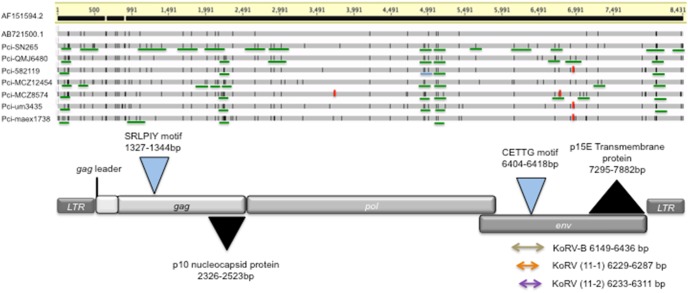
Alignment of modern and museum koala retrovirus sequences, showing positions of proviral genes and proteins. Upper Panel: Character states matching the reference sequence (AF151794) are indicated in light grey, while mismatches (position 312) or polymorphisms (all other positions) are shown as black hatch marks. The infectious clone KV522 (AB721500) is the first sequence below the reference. The aligned sequences all display open reading frames for viral *gag*, *pol*, and *env* regions, except that polymorphisms at three positions in the museum samples code for a stop codon that would disrupt an open reading frame; these are indicated by red hatch marks. Green lines represent polymorphisms that could be placed in phase in overlapping sequence reads. Lower Panel: The coded proteins are indicated, following the divisions proposed by Hanger et al. (2000) relative to the polymorphism alignment. The positions of the SRLPIY domain potentially involved in viral infectivity of the GAG protein and p10 domain (Gag assembly and nuclear export signal, respectively) are indicated. Likewise, the Env motif CETTG, and p15E transmembrane envelope protein are indicated. Regions known to be divergent in Japanese isolates, KoRV-B and KoRV- C, and KoRV-D [12], [40], are indicated by orange, purple, and green arrows, respectively.

Sequences were separated based on their index sequence at the National High-Throughput DNA Sequencing Center, University of Copenhagen, Denmark. The programs cutadapt v1.2 and trimmomatic [21], [22], respectively, were used to remove adaptor sequences and poorly sequenced reads. After trimming, reads that were shorter than 20 bp were excluded from further analyses. Reads were mapped to the KoRV full genome reference sequence (NCBI: AF151794) using BWA version 0.6.2 [23] with default parameters. The resulting SAM files were further processed with samtools [24] and picard (http://picard.sourceforge.net) for sorting and removal of clonality, respectively. The Perl script mapDamage was run on the museum data using the default settings to determine the percentage of DNA damage present, before SNP calling [25]. Variant call analysis was performed using VarScan 2.2.3 with the following settings -min-coverage 8, -min-var-freq 0.01, and -p-value 5e-02 [26]. The resulting variants were further curated using Geneious 6.0.4 for visualization. Negative control reads were also compared to the reference KoRV sequence. The 5′ and 3′ LTRs were distinguished from each other by examining sequences adjacent to the LTR sequence for genomic flank sequences or for KoRV sequence (*gag* leader or *env*). The 5′LTR is preceded by a koala genomic flank and followed by a KoRV *gag* leader, while the 3′LTR is preceded by KoRV *env* and followed by a koala genomic flank. Where possible, LTR sequences that also included a KoRV non-LTR sequence or a koala genomic flanking sequence were used to distinguish between 5′ and 3′ LTR polymorphisms (Figure 1). Consensus sequences generated were deposited in GenBank (accession numbers KF786280–KF786286). Illumina reads mapping to KoRV for each koala were deposited in the NIH Short Read Archive (SRP03960187947).

Integration sites were identified in sequence reads that contained 5′ or 3′ LTR sequences extending into non-KoRV sequences. To examine whether proviral integration sites identified in the ancient samples were present among modern koalas, koala genomic sequences flanking the integration sites found by hybridization were queried against sequences flanking the integration sites of six modern koalas (three northern and three southern koalas, Table 1) that had been generated using a different method (Ishida et al. manuscript in preparation). Integration site sequences were also queried against a koala (Pci-SN404) whole genome sequence generated using one-sixteenth of a PicoTiterPlate of 454 GS-FLX+ Technology (Roche Applied Science) following standard protocols.

### Statistical Analyses and Tests of Selection

For non-synonymous polymorphisms, a “radical” change was defined as a mutation that produces a negative score in both BLOSUM62 and BLOSUM90 substitution matrices. Associations between variables were examined using a 2×2 contingency table, testing for significance using Fisher’s exact test implemented in GraphPad (graphpad.com/quickcalcs/contingency1.cfm). The number of synonymous and non-synonymous substitutions was determined, and the Nei-Gojobori method [27] was used to determine the proportion of synonymous substitutions per synonymous sites and the proportion of non-synonymous substitutions per non-synonymous sites. MEGA, version 5.2 [28] was used to estimate Tajima’s D, and to implement the codon-based Z-test for selection and the codon-based Fisher’s exact test of selection. These were determined for the concatenated KoRV codons of *gag*, *pol*, and *env*, and for each of the three separately. Bonferroni correction for multiple hypothesis testing divided a p value of 0.05 by the number of hypotheses tested.

The *dN/dS* ratio provides an indicator of the selective pressures that acted upon a gene, with low values indicating purifying selection and increases in values indicating relaxation of constraint or positive selection. To account for the different phase of polymorphisms at the same site we generated an individual sequence for each different phase of a polymorphism and analyzed all of the individual sequences. For the modern koala, available sequences were long enough for phase to be determined for many (but not all) polymorphisms (Figure 1, positions underlined in green). For historical samples, sequence lengths were short, and the phase of polymorphisms could only rarely be determined. To test for this signature of selection in this dataset, we calculated *dN*/*dS* using two different approaches: the GA-Branch and FUBAR methods [29]. In the first case estimates were obtained using a fixed tree topology generated by the Neighbor-joining method. The nucleotide model was specified as GTR; otherwise, the default GA-Branch configuration was used. This dataset, which compares all identified polymorphisms (even if they are not present in the consensus sequence) against the modern sequence, was also analyzed using the Z-test for selection and Tajima’s D.

### Identification of Protein Domains and Functional Residues, and Protein Modeling

The corresponding amino acid sequences were subjected to domain identification analysis using the Conserved Domains Database (CDD) from NCBI. We also examined whether any of the observed polymorphisms alter amino acid residues of known function using the Conserved Features/Sites option of the CDD database.

To examine the structural characteristics of KoRV variants, we predicted their three-dimensional structures using the SWISS-MODEL server [30]. Only models with high statistical support (high reliability score as defined by QMEAN4 values) [31] were considered for further analyses. Using this strategy we were able to reliably model several regions corresponding to different domains of all three viral polypeptides (Gag, Pol, and Env). Pairwise structural alignments and structural superimposition were performed using the DaliLite server [32]. Models and Figures were drawn using Pymol (DeLano Scientific).

### Transcription Binding Factor Site Analysis

The long terminal repeat polymorphic sequences for each koala were analyzed for putative transcription binding domains using MatInspectror software (Genomatix, Munich). The default core similarity and matrix similarity greater than 0.8 were employed as the selection criteria.

## Results

### Hybridization Capture and Sequencing of KoRV

DNA was extracted in an ancient DNA dedicated facility from 10 museum skins from southern (*n* = 2) or northern (*n* = 8) Australian koalas, which had been collected as long as 130 years ago (Table 1). In separate facilities, modern DNA was extracted from blood samples of zoo and free-ranging wild koalas (Table 1). Illumina libraries were prepared from all museum koala DNA extracts (in an ancient DNA facility), and from one sample of modern DNA from an adult northern koala 14 years old, Pci-SN265 (“Mirra-Li”, studbook number 265 from the Zoo Vienna, Austria). In order to process all samples in a single next-generation sequencing run, each library was tagged with a distinct index sequence. Baits for hybridization were generated covering the entire KoRV genome, which was amplified in thirty-eight fragments, each ca. 500 bp, from four koalas representing northern (Pci-SN404) and southern (Pci-SN106, 142, 157) koala KoRV diversity (Table 1). Equimolar amounts of index samples were pooled and applied to KoRV baits bound to streptavidin beads for in-solution hybridization capture. The enriched koala libraries were then sequenced using an Illumina MiSeq. After sequencing, a bioinformatics routine used the distinct index sequence tags to separate sequences by individual. Sequences were screened for quality and reliability before being aligned to KoRV reference genomes (GenBank accession number AF151794, AB721500).

**Figure 2:**
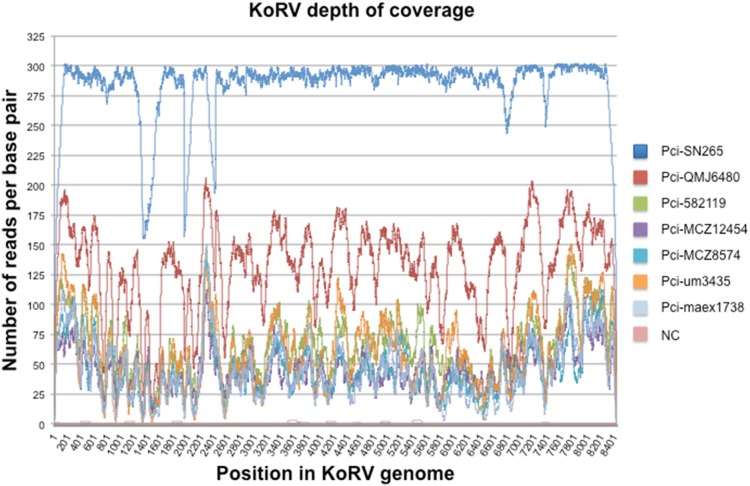
Hybridization capture sequence coverage across the KoRV genome for modern and museum koala samples. The sequence coverage is shown for each nucleotide position numbered as in the KoRV reference genome (AB721500). Results are shown for 1 modern (Pci-SN265) and 6 museum koala samples. Mapping of results for a negative control (NC) are also shown. Each sample is color-coded.

Full coverage of the KoRV genome was obtained from six of the northern Australian museum specimens and for Pci-SN265 (Figure 1); museum specimens of two additional northern and two southern koalas were not successful. Among the six successful historical samples, KoRV-specific sequences represented 2.5% to 41% of the total number of reads, comparable to enrichment rates previously reported for ancient DNA. Negative controls demonstrated only sporadic matches to KoRV (Figure 2). Such sporadic reads are observed in hybridization capture experiments [33] and may reflect index sequence errors (misassignment) or PCR jumping causing exchange between sample index and control [34]. However, the profile of coverage was randomly dispersed and the number of reads marginal in the negative control. Coverage was consistently far higher at every position for the modern koala (Pci-SN265) than for any of the museum specimens (Figure 2). The historical samples yielded only 20–80% of the coverage of the KoRV genome obtained for the modern sample. Among the museum koalas, the earliest collected sample (Pci-maex1738) had the poorest coverage whereas the most recently collected sample (Pci-QMJ6480) had the highest relative coverage. There was otherwise no obvious correlation between the number of reads obtained and the year the sample was collected.

### KoRV Polymorphisms

For the museum samples, the average read length was ca. 90 bp. This is similar to read lengths reported previously for DNA from archival specimens, which may be degraded as a result of environmental, bacterial, and enzymatic damage [35]–[37], and was shorter than the ca. 135 bp read length for modern sample Pci-SN265. Although similar analyses were conducted on the historical and on the modern koala sequences, prior to assembly the museum specimen datasets were processed using the mapDamage Perl script [25], to account for DNA damage present in ancient DNA. The mapDamage results identified the expected nucleotide misincorporation patterns of cytosine to thymine and guanine to adenine on the 5′ and 3′ end termini, respectively (not shown). However, damage occurred only at a very low frequency of 0.02 to 0.08%, indicating that the damage present would have negligible effects on polymorphism scoring or other analyses. After assembling the reads to the KoRV reference sequence AF151794, polymorphisms were scored if they occurred at a position in 8% or more of the reads for an individual koala [38]. For the *env* gene, four of 20 *env* polymorphisms that had been previously detected by PCR from museum samples were also found in the current dataset [6]. Of the remaining 16, seven could be identified but were not present above the cutoff employed when identifying polymorphisms by the current study. The other nine could not be identified from the data, likely due to insufficient coverage in some koalas for those regions of *env* (Figure 2). Fourteen novel polymorphic sites in the *env* region were identified by hybridization capture that had not been identified in the same museum koalas when previously examined by PCR.

At position 312 a fixed difference as opposed to a polymorphism was present in all koalas relative to the reference AF151794 (Figure 1). Across the modern and archival koalas, a total of 138 KoRV polymorphisms were detected. At each of these polymorphisms, one of the character states matched that of the KoRV reference sequence AF151794. Considering only the character states that differed from the reference, seventy-one of the polymorphic sites were detected in two or more koalas (shared alleles) and sixty-seven were detected only in one koala (private alleles) (Table 2, Table S2). Of 92 polymorphisms in the coding regions, 3 would result in stop codons, of which one was shared across individuals (Figure 1, Table 2 KoRV). Of the remaining coding region polymorphisms, 35 were synonymous and 54 were non-synonymous (Table 2, Table S2).

**Table 2:** Types of KoRV polymorphisms detected across 6 museum specimens of koalas.

	LTRs		gag leader		gag		pol		env		Total	
Sequence length	505 bp		465 bp		1566 bp		3384 bp		1980			
	Shared	Private	Shared	Private	Shared	Private	Shared	Private	Shared	Private	Shared	Private
Indels	0	0	2	0	0	0	0	0	0	0	2	0
Polymorphisms of which:	17	14	9	4	17	12	19	19	12	13	74	64
Non-coding	17	14	9	4	NA	NA	NA	NA	NA	NA	26	18
Coding region of which	NA	NA	NA	NA	17	12	19	19	12	13	48	44
Stop codon	NA	NA	NA	NA	0	0	0	1	1	1	1	2
Synonymous	NA	NA	NA	NA	8	5	9	7	3	3	20	15
Non-synonymous of which:	NA	NA	NA	NA	9	7	10	11	8	9	27	27
Not radical	NA	NA	NA	NA	7	3	5	8	2	3	14	14
Radical	NA	NA	NA	NA	2	4	5	3	**6**	**6**	13	13

LTRs are the long terminal repeats, sequence length is from the KoRV reference sequence AF151794. Private polymorphisms were those detected in one koala, shared polymorphisms in more than one koala; NA is not applicable. Radical changes are atypical amino acid substitutions with negative scores in both BLOSUM62 and BLOSUM90 matrices. Radical mutations in *env* (boldface) were more common (p = 0.040) than in *gag*-*pol*, relative to other amino acid changes.

Functional regions in the viral sequence reported to reduce the infectivity of KoRV when compared to that of GALV were also examined [3]. The CETAG motif in KoRV (CETTG in other gammaretroviruses) is believed to be responsible for viral fusion activity while the *gag* L-domain is believed to affect the release of mature virus. Across KoRVs in the newly sequenced koalas, there were no polymorphic sites in either of these regions (Figure 1). The immunosuppressive domain of the p15E transmembrane protein of retroviral Env exhibited only a single polymorphism, present in Pci-SN265 and Pci-MCZ12454 (Table S2). In the museum specimens, multiple non-synonymous polymorphisms were detected in the nucleocapsid protein region (p10) of the *gag* gene. Using the Conserved Features/Sites function of the CDD database we also determined that none of the amino acid residues of known or inferred function, e.g., DNA binding site of the reverse transcriptase domain in POL or the homotrimer interface in ENV, are polymorphic (data not shown).

Amino acid substitution matrices have been generated by comparing large numbers of proteins to identify non-synonymous mutations that are only rarely observed empirically. These rare amino acid substitutions, termed “radical”, typically involve major physiochemical differences between the two amino acids. We defined a radical change as a mutation that produces a negative score in both BLOSUM62 and BLOSUM90 substitution matrices. Among the non-synonymous polymorphisms observed in the koala, 48% (26/54) of substitutions were defined as radical. The proportion of radical non-synonymous mutations appeared to be higher in *env* than in *gag* or *pol* (Table 2), and this difference was confirmed as significant using Fisher’s exact test (p = 0.0397) comparing radical vs. non-radical non-synonymous substitutions in *env* to those in *gag-pol*. This suggested that selective constraints on *env* may differ from those affecting the other two KoRV coding regions. Across the three coding regions, no other pattern suggestive of an association across variables was evident in the dataset for private vs. shared polymorphisms, non-synonymous vs. synonymous polymorphisms, or radical vs. non-radical amino acid changes (Table 2).

The selective pressure variation among all branches of the KoRV tree estimated by the GA-Branch method suggested that several branches in *gag* (more than 70%) and fewer in *pol* and *env* (60 and 17%, respectively) are under purifying selection (not shown). FUBAR implemented in HyPhy also suggested that only a few codons deviate neutrality (not shown). Similarly, the total distance estimates of dN/dS using the Nei-Gojobori method suggested stronger purifying selection in *gag* than in *pol* and *env* (Table S3). The same trend was observed by the Z-tests for selection and the Tajima’s test of neutrality, with *gag* showing multiple significantly negative dN-dS values and the lowest negative values, respectively (Table S4 and Table S5).

### Comparisons of KoRV Consensus Sequences

The nucleotide consensus sequence (majority character state at every position in an alignment of sequences) was generated for each of the seven successful KoRV-positive koalas. These were compared to the first reported KoRV sequence (AF151794) [7] and to the infectious clone KV522 (AB721500) [39], which themselves are 0.5% divergent, generating an alignment of 9 sequences. Each of the newly generated consensus sequences was more similar to the infectious clone KV522 (99.2–99.5% similarity) versus for AF151794 (99.0–99.2% similarity). All of the koala retroviral consensus sequences from the current study included a 6-bp insertion in the non-coding *gag* leader region, position 651, which is also present in KV522 (Figure 1, Table S2). The six archival sample consensus sequences also shared a 3-bp insertion at position 905 in the *gag* leader region (Table S2) also present in KV522 (AB721500) [39]. The 3 bp insertion could be found in the modern koala (Pci-SN265) as a minority sequence, thus the consensus for this animal lacked the 3 bp insertion (Figure 1). Thus, in contrast to the museum samples, the modern koala had an underrepresentation (16.8%) of the 3 bp insertion variant. In addition, the deletion itself is polymorphic representing 1–3 bp deletions though the 3 bp deletion is the most common and therefore represented in the consensus sequence generated.

The alignment of nine sequences was also examined for signatures of selection (or neutrality), for *gag*, *pol*, and *env*, and for all three codon sets concatenated. The Nei-Gojobori method was used to estimate synonymous and non-synonymous mutation rates for each pair of sequences. Codon-based Fisher’s exact tests of selection found no evidence of positive selection in any of the pairwise comparisons for any of the coding regions (not shown). Codon based Z-tests of selection (Table 3) suggested that among the coding regions purifying selection may be more pronounced in *gag*, with significant purifying selection detected in eight of the pairwise comparisons (although these would not be significant after Bonferroni correction). The *pol* coding region appeared to be under weaker purifying selection, with negative values significant (before Bonferroni correction) for only 2 comparisons, while *env* comparisons yielded both positive and negative estimates consistent with neutrality (none significant). Tajima’s D was calculated using the same nine KoRV sequences (Table 4), for each coding region separately or all three combined. A negative value would be consistent with purifying selection. However, although *gag* had the most negative value, none of the values for Tajima’s D were extreme, thus there were no significant deviations from neutrality. The consensus sequence and total polymorphism data yielded consistent results with respect to selective pressures on KoRV.

**Table 3:** Codon based Z tests of selection.

		1	2		3	4	5	6	7	8	9
	*gag* (520 codons used)										
1	KoRV_AF151794		−1,690		−2,816	−1,512	−2,287	−1,216	−1,898	1,168	−0,813
2	KoRV_AB721500	0,094			−2,164	−1,119	−1,362	−1,549	−1,175	−0,205	−0,818
3	Pci-SN265	**0,006**	**0,032**			−2,305	−1,383	−1,533	−1,813	−1,676	−2,102
4	Pci-QMJ6480	0,133	0,266		**0,023**		−2,142	−1,762	−1,937	−0,369	−0,789
5	Pci-582119	0,024	0,176		0,169	**0,034**		−2,133	−2,330	−1,491	−1,795
6	Pci-MCZ_12454	0,226	0,124		0,128	0,081	**0,035**		−1,841	−0,754	−1,719
7	Pci-MCZ8574	0,060	0,242		0,072	0,055	**0,021**	0,068		−0,865	−1,343
8	Pci-um3435	0,245	0,838		0,096	0,713	0,139	0,452	0,389		0,283
9	Pci-maex1738	0,418	0,415		**0,038**	0,431	0,075	0,088	0,182	0,778	
	*pol* (1127 codons used)										
1	KoRV_AF151794		−1,175		−2,084	−0,269	−1,521	−0,162	0,370	−1,407	−0,654
2	KoRV_AB721500	0,242			−1,523	−0,950	−0,855	−1,099	0,384	−1,390	−0,807
3	Pci-SN265	**0,039**	0,130			−1,489	−0,949	−2,657	−1,449	−1,158	−1,827
4	Pci-QMJ6480	0,788	0,344		0,139		−1,188	−1,163	−0,904	0,339	−1,340
5	Pci-582119	0,131	0,394		0,345	0,237		−1,798	−0,720	−1,400	−0,942
6	Pci-MCZ_12454	0,871	0,274		**0,009**	0,247	0,075		−0,816	−1,710	−0,881
7	Pci-MCZ8574	0,712	0,701		0,150	0,368	0,473	0,416		−1,369	0,469
8	Pci-um3435	0,162	0,167		0,249	0,735	0,164	0,090	0,174		−1,192
9	Pci-maex1738	0,515	0,421		0,070	0,183	0,348	0,380	0,640	0,236	
	*env* (659 codons used)										
1	KoRV_AF151794		1,437		−0,968	−0,933	0,312	0,223	0,805	−1,123	−0,937
2	KoRV_AB721500	0,153			−1,334	−0,924	0,013	−0,204	0,562	−1,351	−0,731
3	Pci-SN265	0,335	0,185			0,014	−1,135	−0,748	−0,735	0,844	−0,349
4	Pci-QMJ6480	0,353	0,357		0,989		−0,516	−0,744	0,019	1,245	−0,750
5	Pci-582119	0,755	0,990		0,259	0,607		−0,161	−0,416	−0,205	−0,209
6	Pci-MCZ_12454	0,824	0,839		0,456	0,458	0,873		0,187	−0,333	−1,305
7	Pci-MCZ8574	0,422	0,575		0,464	0,985	0,678	0,852		−0,209	0,227
8	Pci-um3435	0,264	0,179		0,400	0,215	0,838	0,740	0,835		0,018
9	Pci-maex1738	0,351	0,466		0,727	0,455	0,835	0,194	0,821	0,986	
	Combined (2306 codons used)										
1	KoRV_AF151794			−1,739	−3,149	−1,657	−2,268	−0,759	−0,829	−1,096	−1,254
2	KoRV_AB721500	0,085			-2,619	−1,809	−1,325	−1,721	−0,308	−1,623	−1,258
3	Pci-SN265	**0,002**		**0,010**		−2,512	−1,961	−2,918	−2,125	−1,508	−2,508
4	Pci-QMJ6480	0,100		0,073	**0,013**		−2,433	−2,057	−1,941	0,533	−1,743
5	Pci-582119	**0,025**		0,188	0,052	**0,016**		−2,282	−1,961	−1,968	−1,708
6	Pci-MCZ_12454	0,449		0,088	**0,004**	**0,042**	**0,024**		−1,478	−1,646	−1,955
7	Pci-MCZ8574	0,409		0,758	**0,036**	0,055	0,052	0,142		−1,593	−0,510
8	Pci-um3435	0,275		0,107	0,134	0,595	0,051	0,102	0,114		−0,816
9	Pci-maex1738	0,212		0,211	**0,013**	0,084	0,090	0,053	0,611	0,416	

The test statistic dN-dS is shown above the diagonal. dN and dS are the values of non-synonymous and synonymous substitutions per site, respectively. The Nei-Gojobori method was used to calculate synonymous and nonsynonymous substitutions. The probability of rejecting the null hypothesis of strict-neutrality (dN  =  dS) is shown below the diagonal). Values of P less than 0.05 are highlighted in bold. Values were not significant after Bonferroni correction. The variance of the difference was computed using the bootstrap method (500 replicates). Numbers listed for columns represent the same KoRV sequences numbered in the rows. The first two KoRV sequences are from GenBank; the other KoRVs are consensus sequences from the current study.

**Table 4:** Estimates of Tajima’s D*.

	m	S	ps	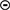	π	D
*gag*	9	36	0.023003	0.008464	0.007419	−0.623145
*pol*	9	37	0.010934	0.004023	0.004145	0.153741
*env*	9	22	0.011111	0.004088	0.004097	0.010077
all	9	95	0.01371	0.005045	0.004871	−0.177721

*The analysis involved 9 KoRV sequences. Codon positions included were 1st+2nd+3rd. All positions containing gaps or missing data were eliminated. There were 1565 positions for *gag*, 3384 for *pol*, 1980 for *env*, and 6929 positions for all (concatenated coding sequences) in the final dataset. Abbreviations: m  =  number of sequences; S = Number of segregating sites; ps = S/m; Θ = ps/a1; π = nucleotide diversity; D = Tajima test statistic.

### KoRV-B and J

A recent study of koalas from Los Angeles Zoos identified a KoRV variant, designated KoRV-B, which has greater virulence than previously characterized KoRV, and which was present in a subset of zoo koalas [12]. Hybridization capture should enrich sequences, such as those of KoRV-B, that are somewhat divergent from the KoRV sequence used as bait. We therefore screened the novel next generation sequencing data, searching for the sequence of KoRV-B at the junction where KoRV-B diverges from the KoRV reference sequences in the *env* region. The divergent region of KoRV-B *env* was not detected in any of the ancient koalas, suggesting that KoRV-B may have evolved recently as a variant of KoRV. However in the modern koala Pci-SN265, sequences matching KoRV-B were detected for some (but not all) of the regions within the *env* gene that characterize KoRV-B (Figure S1).

Three other variants of KoRV have recently been described among zoo koalas in Japan, tagged as clones 11-1, 11-2, and 11-4 [11], [40]. Clone 11-1 has been designated KoRV-D, clone 11-2 has been designated KoRV-C, and clone 11-4 has been designated KoRV-J. The three recently identified KoRV clones differ mainly in variable region A of the *env* gene that is involved in retroviral receptor determination and recognition [11], [41]. Our novel sequences were screened for each of the KoRV variants reported in the Japanese zoo koalas. Sequences similar to those of KoRV-C and KoRV-D were identified in the modern koala Pci-SN265 but not in any of the museum samples (Figure S2 A and B) [40]. Sequences related to KoRV-J were not identified in any of the novel reads, whether from the modern or museum samples.

### Potential Effects of KoRV Polymorphisms on Protein Structure

**Figure 3:**
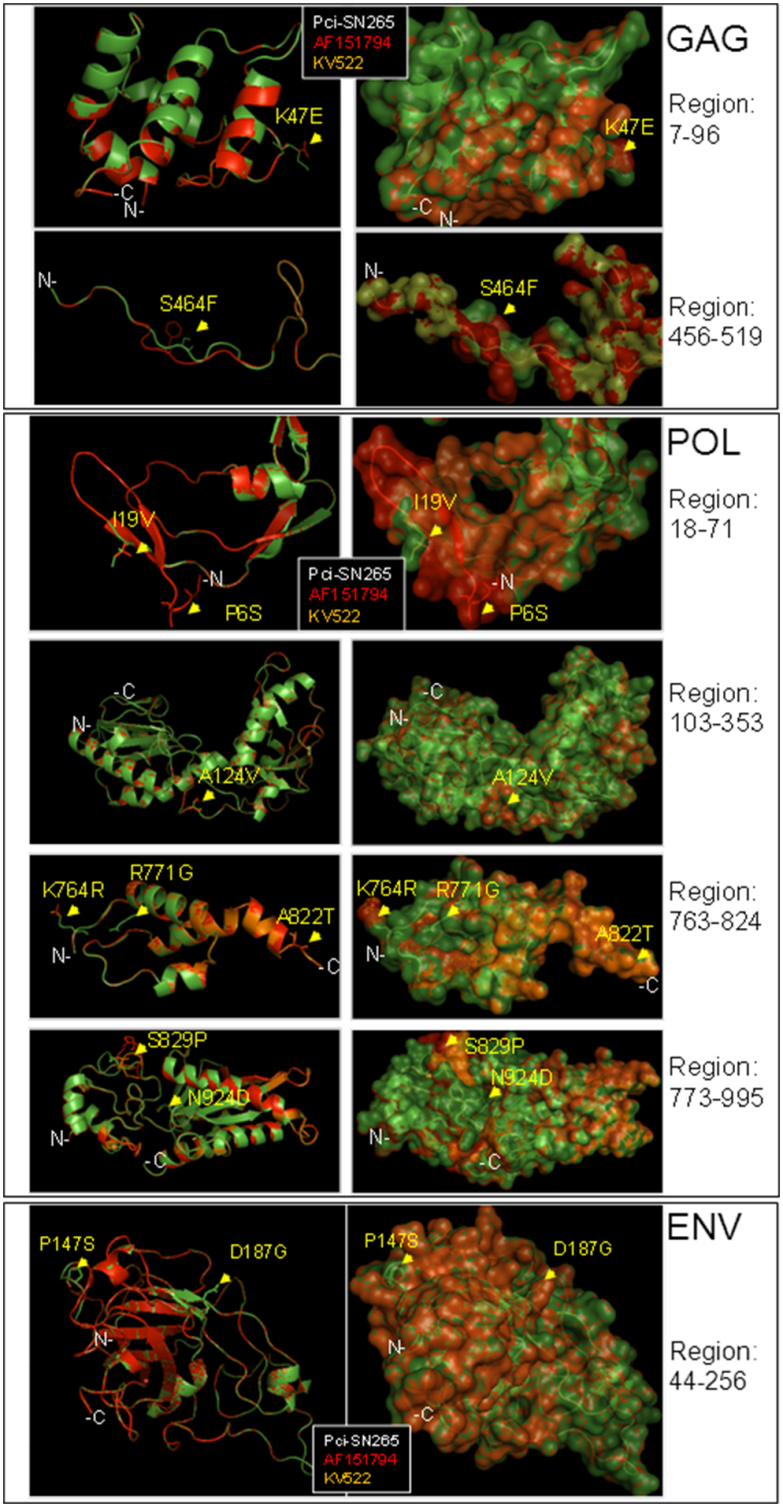
Structural superimpositions of Pci-SN265 (green), AF151794 (red), and KV522 (gold) KoRV Gag, Pol, and Env protein structures, demonstrating the overall similarity of the structures. Amino acid variations across these three sequences are mapped on the protein models (arrows). The structural differences predicted are attributed to changes in the polarity, charge, and atom conformations. The models are shown in cartoon (left panels) and semi-transparent surface (right panels) representations. The atoms of the variable amino acid residues are shown in line representations to view the side chains. In all comparisons the Pci-SN265 consensus sequence was used as the reference.

Variants present below a cutoff of 8% of relevant Illumina reads were not considered to represent confirmable polymorphisms. Those that appeared at a higher frequency than this cutoff likely represent common variants rather than mutations within a single provirus. We examined the effects of the non-synonymous polymorphisms on the protein structure of KoRV by generating three-dimensional models for Gag, Pol, and Env protein fragments. First, we sought to identify whether amino acid differences present across modern sequences of KoRV led to major structural differences. Sequences included in the comparisons of modern KoRV were the original KoRV isolate AF151794 and infectious clone KV522 (AB721500), which differ by 0.5% at the nucleotide. In these comparisons, our consensus KoRV sequence from the modern koala Pci-SN265 served as the reference (the amino acid residue in Pci-SN265 is always the first listed in each substitution). When superimposed on the structure of Pci-SN265, the structures of AF151794 and KV522 showed minor localized changes affecting the polarity, charge, or local protein conformation (Figure 3). Specifically, in the Gag protein mutations K47E and S464F alter the local charge and the local protein conformation, respectively (Figure 3). In the Pol protein, mutations P6S, A124V, K764R, R771G, and N924D between the Pci-SN265 and AF151794 had only minor effects on the overall topology of the structure. In Pol, mutations I19V, A822T, and S829P altered the local conformation of the Pci-SN265 and KV522 relative to the AF151794 structure by changing a surface residue to a buried one (I19V) and changing two partially buried residues to surface ones (A822T and S829P) (Figure 3). Only two positions in the Env protein could be structurally modeled (P147S, D187G). Both of these were radical substitutions that changed buried amino acids to exposed ones (Figure 3). Both of these changes were located away from the putative receptor-binding region, as this has been defined in [6].

**Figure 4:**
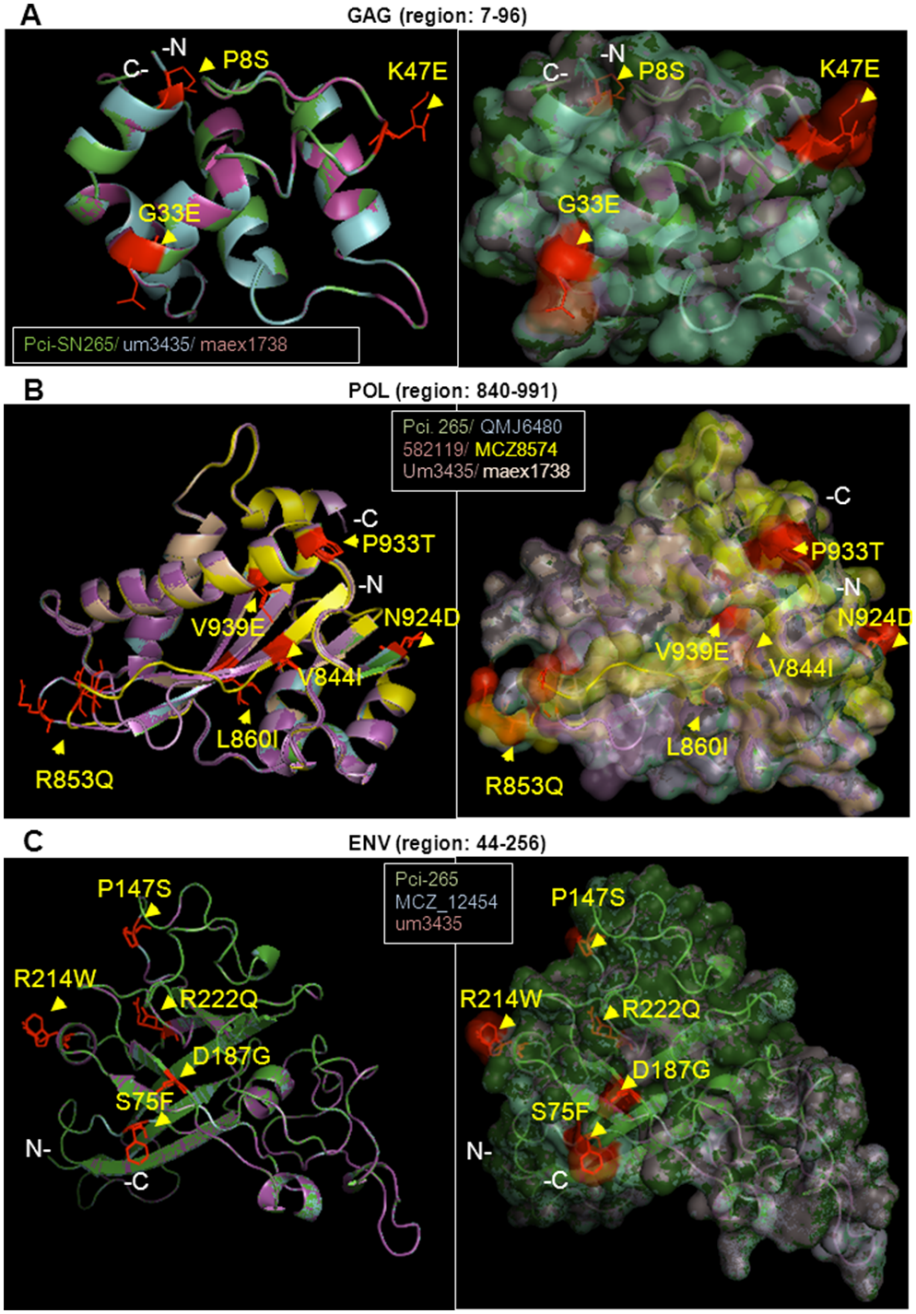
The effects of historical KoRV polymorphisms on protein structure. Superimpositions are shown between the present day consensus KoRV (Pci-SN265) protein structure and ancient KoRV variants. Amino acid variations between these sequences mapped on the protein models are shown in red and with arrows. The models are shown in cartoon ribbon representations (left panels) and as semi-transparent surfaces (right panels). The atoms of the variable amino acid residues are in line representations to view the side chains. In all comparisons the Pci-SN265 consensus was used as the reference sequence. (A) The model of the Pci-SN265 Gag protein is superimposed with the models of variants found in archival koalas um3435 and maex1738. (B) The model of the Pci-SN265 Pol protein is superimposed with variants found in QMJ6480, 582119, MCZ8574, Um3435, and maex1738. (C) The model of the Pci-SN265 Env protein is superimposed with the model of variants found in MCZ_12454 and um3435. For all three polypeptides, the structural differences predicted are attributed to changes in the polarity, charge, and atom conformations and are largely localized onto flexible loop regions.

Second, non-synonymous polymorphisms in the historical KoRV sequences were examined for predicted changes to the protein structure as compared to the modern consensus sequence Pci-SN265, which again served as a reference sequence (the amino acid residue in Pci-SN265 is always the first listed in each substitution). The effects on polypeptide structures of the non-synonymous polymorphisms present in KoRV for each koala were examined using a composite sequence that contained all of the amino acid differences versus Pci-SN265. This composite sequence would necessarily combine polymorphisms present on different proviral loci. Nonetheless, this composite sequence would be useful in identifying all of the potential disruptions to predicted structure, when the effects of each mutation are considered individually. Several ancient variants were predicted to cause small local fluctuations of the KoRV structure. Specifically positions G33E, K421E, Q429K, and S464Y are predicted to alter the local charge at the Gag surface resulting in deviated conformations (Figure 4A, Figure S3). In the Pol protein, positions S514R, F396Y, A685S, and Y676N exchange a buried residue for a surface one, resulting in topological differences (Figure 4B, Figure S4). Additionally, in the Pol protein major conformational changes were predicted to occur at ancient variants R853Q, P933T, V939E, and T1014I. Lastly, two ancient variants S75F and R214W found in the Env protein were predicted to have major structural effects (Figure 4C, Figure S5). Both of these changes are located away from the putative receptor binding region as this has been defined in [6]. It is important to note that the other character state found at each of the polymorphic sites in the ancient koalas matched the character state present in the reference sequence. Thus, despite the presence of these polymorphisms and their modeled effects on proteins, KoRV overall has remained stable in sequence and structure over time.

### LTR and Integration Site Diversity

Multiple polymorphisms were observed in the long terminal repeats (LTRs) that serve as promoter and terminator of the retroviral transcription process (Figure 1). MatInspector (Genomatix) was used to examine U3, R, and U5 regions of the LTRs for sequences matching transcription factor binding sites (TFBs) and for disruptions of TFBs by polymorphisms. Polymorphic sites from the next generation sequencing data were placed in phase when possible (Figure 1). This analysis of the LTRs revealed the presence of 82 putative TFBs (Table S3). Six of these had been referred to by Hanger et al. (2000), including a TATA box, CCAAT retroviral signal, and C-type poly-A signal (Table S6). None of the polymorphisms detected would have disrupted the previously predicted TFBs. However 20 additional predicted TFBs would be generated by the various polymorphisms.

Sequences at the 5′ and 3′ end of the KoRV genome often extended beyond the proviral integration sites into the host genomic flank. Shared integration sites among koalas would be strong evidence that a given locus represents an endogenized retrovirus, since the chance that two proviruses would independently integrate into the same locus is minuscule [42]. Four hundred twenty nine 5′ flanks and three hundred thirty one 3′ flanks were identified across all the koalas tested (Table S7). Thirty-two 5′ and twenty-three 3′ flanking sequences were shared by two or more koalas, representing 7.5% and 7% of the total respectively (Table S4).

The sequences flanking the integration sites were queried against flanks found in six modern koalas, which had been detected by other methods (Ishida et al., in preparation). Eight of the integration sites found by hybridization capture were also identified in one or more of six modern koalas tested (Table S7). The sequences flanking KoRV were also queried against whole genome sequences from a single koala, generated from one-sixteenth plate GS-FLX shotgun sequence (Ishida et al., in preparation). Two flanking sequences had matches among the GS-FLX results. One of the flanking sequences matched 45 of the next-generation sequences, suggesting that this KoRV provirus had integrated in a repetitive element of the koala genome. The other matching flank sequence was detected only once. This sequence was KoRV negative at the locus, with the host genomic flank on the other side of the provirus evident in the sequence.

## Discussion

Hybridization capture using archival samples has been used to efficiently sequence mitogenomes [15], bacterial genes [43] and low copy number genomically integrated viruses [44]. Here we use hybridization capture to generate sequences at high coverage across the full length of KoRV from both museum samples and modern genomic DNA. Information on both the provirus and its integration sites was obtained simultaneously, providing information on ca. 130 years of KoRV evolution. Limited variation was detected across the entire proviral genome including the LTRs. A previous study had examined *env* from several of the same samples used in this study. Using PCR and GS FLX sequencing, 20 polymorphisms and one fixed difference had been reported for *env* between museum samples and reference sequence AF151794 [6]. Of these 20, only 4 polymorphisms were also identified by the current study. However, 7 of the remaining 16 polymorphisms were also detectable in the current dataset, but at levels below the 8% threshold used to screen for polymorphisms. This may reflect the bias introduced by PCR based approaches to ancient DNA, where molecules amplified in the earlier cycles (of which there are few to begin with) may come to dominate in the pool of sequences. This is particularly true for historical DNA where 60 or more cycles have been used to generate templates. In contrast, hybridization capture does not initially rely on PCR in enriching the target from the library. Library primers are used post enrichment to generate sufficient template for sequencing. However, all templates have the sequences targeted by the primers, and a lower cycle number is used (7–30 cycles), which should yield a less biased data set. The drawback, common to PCR and hybridization capture, is that very low frequency polymorphisms may not be scored above background error and DNA damage levels, though this is anticipated to be a lesser problem with modern DNA than ancient DNA (which has a lower number of templates). Variation in coverage also influenced polymorphism scoring in the hybridization capture data; this had a larger impact on the historical samples that generally have lower coverage (Fig. 2). For example 6 of the *env* polymorphisms not identified were likely due to low coverage in *env* for the poorest performing sample, Pci-maex1738. However, hybridization capture of all samples identified 14 novel polymorphisms not previously detected by PCR, including two novel polymorphisms in the poorest performing sample Pci-maex1738. Increasing the depth of coverage is possible with hybridization capture, whereas removing bias from PCR based approaches is not. Thus, the ease, coverage and lower expense of hybridization capture provide advantages over PCR based approaches.

The polymorphisms in the *gag*, *pol* and *env* coding regions did not display any evident differences in the proportions of private versus shared alleles and/or in terms of synonymous versus non-synonymous mutations. However, the number of radical versus non-radical amino acid was significantly different across the three coding regions. The relative number of radical mutations, those corresponding to large physiochemical differences between the amino acids, was significantly elevated in the *env* coding region when compared to *gag*-*pol* coding regions. The higher proportion of radical changes in Env could potentially reflect either anti-viral immune pressure on the exposed portions of the Env proteins or avoidance of receptor interference [45]–[49].

However, none of the non-synonymous substitutions altered functional regions of the respective proteins reported as being critical for infection or replication previously reported [3] or altered any of the residues that have been functionally characterized in other viruses based on the CDD database (NCBI). The latter results imply that negative selection could be responsible for the conservation of these sites although statistically, deviation from neutral evolution was not observed.

Most tests of selection suggested that the evolution of KoRV does not deviate greatly from neutrality. An alignment of two KoRV sequences from GenBank with seven KoRV consensus sequences derived from the novel data did suggest a trend for purifying selection to have played a stronger role in *gag* and to have a reduced role in *env*. A weak trend was evident in calculations of Tajima’s D, and was also suggested by codon-based Z-tests, although these were not significant after Bonferroni correction. An elevated number of non-synonymous changes in the Gag protein may potentially suggest that anti-viral proteins such as TRIM5alpha are acting on KoRV. Evidence for such selective pressure has been studied for TRIM5alpha itself [50]. Although these analyses indicate relaxation of constraints overall, purifying selection may have shaped and conserved particular structural and functional elements.

The LTR region enrichment also resulted in retrieval of viral integration sites. Only ca. 7% of the integration sites were found in two or more koalas which suggests fixation of specific KoRV integrations is not very advanced in koalas even where KoRV has been present the longest such as Queensland. The large number of unique integrations is consistent with previously reported results for Southern blot hybridization based on *pol* and *env* genes, which suggested that KoRV integration sites were quite variable across individual koalas [51]. Although the hybridization capture method would potentially capture both endogenous and exogenous proviruses, the presence of proviruses at the same locus in more than one koala would indicate that at least some of the sequences obtained are from endogenous retroviruses.

We found no evidence for KoRV-B in any of the historical koalas, although partial KoRV-B receptor sequences were identified in the modern koala Pci-SN265. The modern koala was born in the Houston Zoo, Texas and had a complex pedigree and transfer history including transfer among American and European institutions. Thus, exposure to KoRV-B infected individuals may have been possible although the exact source of infection cannot be determined. Alternatively, KoRV-B may be more widespread in captive koalas than previously estimated. However, the absence of KoRV-B in the historical datasets would be consistent with a recent emergence of this variant. By contrast, two of three KoRV variants described from koalas in Japanese zoos were also detected in the modern koala Pci-SN265. The absence of KoRV-J sequences in the museum koalas is consistent with a recent origin of these sequences.

Overall, our results suggest that for ca. 130 years, the majority of KoRV proviruses have remained conserved with one of the character states at each ancient polymorphism matching that of modern KoRV. Considering the potential pathological effects of modern KoRV, its historical genomic and structural stability suggests that koalas have suffered long term negative health impacts in populations where KoRV has occurred. It also suggests that fitness may eventually decrease in koala populations in southern Australia where KoRV appears to be emerging.

## Supporting Information

Figure S1 **Alignment of hybridization capture sequences to koala retrovirus B (KoRV-B) isolate Br2–1CETTG.**
(PDF)

Figure S2 **Alignment of hybridization capture sequences to KoRV isolates identified in Japanese zoo koalas: clone 11–1 (panel A) and 11–2 (Panel B).**
(PDF)

Figure S3 **Superimpositions between the modern KorV (Pci-SN265-green) protein structure and its historical variants show the overall similarity of the structures of the Gag protein.**
(PDF)

Figure S4 **Superimpositions between the modern KoRV (Pci-SN265-green) protein structure and its historical variants show the overall similarity of the structures of the Pol protein.**
(PDF)

Figure S5 **Superimpositions between the modern KoRV (Pci-SN265-green) protein structure and its historical variants show the overall similarity of the structures of the Env protein.**
(PDF)

Table S1 **Koala retrovirus (KoRV) primers.**
(PDF)

Table S2 **KoRV variable sites in modern and historic koalas.**
(PDF)

Table S3 **Estimates of the overall dn and ds distance and the dn/ds ratios.**
(PDF)

Table S4 **Estimates of Tajima’s D.**
(PDF)

Table S5 **Codon based Z tests of selection.**
(PDF)

Table S6 **Variation in putative transcription factor binding sites in KoRV LTRs.**
(PDF)

Table S7 **KoRV flanking sequences common to two or more koalas.**
(PDF)

## References

[1] Tarlinton R, Meers J, Young P (2008) Biology and evolution of the endogenous koala retrovirus. Cell Mol Life Sci 65: 3413–3421. 18818870 10.1007/s00018-008-8499-y PMC11131744

[2] Hunter P (2010) The missing link. Viruses revise evolutionary theory. EMBO Rep 11: 28–31. 20033091 10.1038/embor.2009.267 PMC2816630

[3] Oliveira NM, Satija H, Kouwenhoven IA, Eiden MV (2007) Changes in viral protein function that accompany retroviral endogenization. Proc Natl Acad Sci U S A 104: 17506–17511. 17959780 10.1073/pnas.0704313104 PMC2077286

[4] Cornelis G, Heidmann O, Degrelle SA, Vernochet C, Lavialle C, et al (2013) Captured retroviral envelope syncytin gene associated with the unique placental structure of higher ruminants. Proc Natl Acad Sci U S A 110: 828–837. 10.1073/pnas.1215787110 PMC3587263 23401540

[5] Kewitz S, Staege MS (2013) Expression and Regulation of the Endogenous Retrovirus 3 in Hodgkin’s Lymphoma Cells. Front Oncol 3: 179. 23847767 10.3389/fonc.2013.00179 PMC3706881

[6] Avila-Arcos MC, Ho SY, Ishida Y, Nikolaidis N, Tsangaras K, et al (2013) One hundred twenty years of koala retrovirus evolution determined from museum skins. Mol Biol Evol 30: 299–304. 22983950 10.1093/molbev/mss223 PMC3548305

[7] Hanger JJ, Bromham LD, McKee JJ, O’Brien TM, Robinson WF (2000) The nucleotide sequence of koala (*Phascolarctos cinereus*) retrovirus: a novel type C endogenous virus related to Gibbon ape leukemia virus. J Virol 74: 4264–4272. 10756041 10.1128/jvi.74.9.4264-4272.2000 PMC111943

[8] Tarlinton R, Meers J, Hanger JJ, Young P (2005) Real-time reverse transcriptase PCR for the endogenous koala retrovirus reveals an association between plasma viral load and neoplastic disease in koalas. Journal of general virology 86: 783–787. 15722540 10.1099/vir.0.80547-0

[9] Simmons GS, Young PR, Hanger JJ, Jones K, Clarke D, et al (2012) Prevalence of koala retrovirus in geographically diverse populations in Australia. Aust Vet J 90: 404–409. 23004234 10.1111/j.1751-0813.2012.00964.x

[10] Stoye JP (2006) Koala retrovirus: a genome invasion in real time. Genome Biol 7: 241. 17118218 10.1186/gb-2006-7-11-241 PMC1794577

[11] Shojima T, Yoshikawa R, Hoshino S, Shimode S, Nakagawa S, et al (2013) Identification of a novel subgroup of Koala retrovirus from Koalas in Japanese zoos. J Virol 87: 9943–9948. 23824806 10.1128/JVI.01385-13 PMC3754120

[12] Xu W, Stadler CK, Gorman K, Jensen N, Kim D, et al (2013) An exogenous retrovirus isolated from koalas with malignant neoplasias in a US zoo. Proc Natl Acad Sci U S A 110: 11547–11552. 23798387 10.1073/pnas.1304704110 PMC3710800

[13] Briggs AW, Good JM, Green RE, Krause J, Maricic T, et al (2009) Targeted retrieval and analysis of five Neandertal mtDNA genomes. Science 325: 318–321. 19608918 10.1126/science.1174462

[14] Maricic T, Whitten M, Pääbo S (2010) Multiplexed DNA sequence capture of mitochondrial genomes using PCR products. PLoS One 5(11): e14004. 21103372 10.1371/journal.pone.0014004 PMC2982832

[15] Mason VC, Li G, Helgen KM, Murphy WJ (2011) Efficient cross-species capture hybridization and next-generation sequencing of mitochondrial genomes from noninvasively sampled museum specimens. Gen Res 21: 1695–1704. 10.1101/gr.120196.111 PMC3202286 21880778

[16] Tsangaras K, Avila-Arcos MC, Ishida Y, Helgen KM, Roca AL, et al (2012) Historically low mitochondrial DNA diversity in koalas (*Phascolarctos cinereus*). BMC Genet 13: 92. 23095716 10.1186/1471-2156-13-92 PMC3518249

[17] Wyatt KB, Campos PF, Gilbert MT, Kolokotronis SO, Hynes WH, et al (2008) Historical mammal extinction on Christmas Island (Indian Ocean) correlates with introduced infectious disease. PLoS One 3(11): e3602. 18985148 10.1371/journal.pone.0003602 PMC2572834

[18] Meyer M, Kircher M (2010) Illumina sequencing library preparation for highly multiplexed target capture and sequencing. Cold Spring Harbor Protocols doi:10.1101/pdb.prot5448. 10.1101/pdb.prot5448 20516186

[19] Rozen S, Skaletsky H (2000) Primer3 on the WWW for general users and for biologist programmers. Methods Mol Biol 132: 365–386. 10547847 10.1385/1-59259-192-2:365

[20] Hanke M, Wink M (1994) Direct DNA sequencing of PCR-amplified vector inserts following enzymatic degradation of primer and dNTPs. Biotechniques 17: 858–860. 7639844

[21] Lindgreen S (2012) AdapterRemoval: easy cleaning of next-generation sequencing reads. BMC Res Notes 5: 337. 22748135 10.1186/1756-0500-5-337 PMC3532080

[22] Martin M (2011) Cutadapt removes adapter sequences from high-throughput sequencing reads. EMBnet journal 17: 10–12.

[23] Li H, Durbin R (2009) Fast and accurate short read alignment with Burrows-Wheeler transform. Bioinformatics 25: 1754–1760. 19451168 10.1093/bioinformatics/btp324 PMC2705234

[24] Li H, Handsaker B, Wysoker A, Fennell T, Ruan J, et al (2009) The Sequence Alignment/Map format and SAMtools. Bioinformatics 25: 2078–2079. 19505943 10.1093/bioinformatics/btp352 PMC2723002

[25] Ginolhac A, Rasmussen M, Gilbert MT, Willerslev E, Orlando L (2011) mapDamage: testing for damage patterns in ancient DNA sequences. Bioinformatics 27: 2153–2155. 21659319 10.1093/bioinformatics/btr347

[26] Koboldt DC, Zhang Q, Larson DE, Shen D, McLellan MD, et al (2012) VarScan 2: somatic mutation and copy number alteration discovery in cancer by exome sequencing. Genome Res 22: 568–576. 22300766 10.1101/gr.129684.111 PMC3290792

[27] Nei M, Gojobori T (1986) Simple methods for estimating the numbers of synonymous and nonsynonymous nucleotide substitutions. Mol Biol Evol 3: 418–426. 3444411 10.1093/oxfordjournals.molbev.a040410

[28] Tamura K, Peterson D, Peterson N, Stecher G, Nei M, et al (2011) MEGA5: Molecular Evolutionary Genetics Analysis using Maximum Likelihood, Evolutionary Distance, and Maximum Parsimony Methods. Molecular Biology and Evolution 28: 2731–2739. 21546353 10.1093/molbev/msr121 PMC3203626

[29] Pond SLK, Frost SDW (2005) A genetic algorithm approach to detecting lineage-specific variation in selection pressure (vol 22, pg 478, 2005). Molecular Biology and Evolution 22: 1157–1157. 10.1093/molbev/msi031 15509724

[30] Arnold K, Bordoli L, Kopp J, Schwede T (2006) The SWISS-MODEL workspace: a web-based environment for protein structure homology modelling. Bioinformatics 22: 195–201. 16301204 10.1093/bioinformatics/bti770

[31] Benkert P, Biasini M, Schwede T (2011) Toward the estimation of the absolute quality of individual protein structure models. Bioinformatics 27: 343–350. 21134891 10.1093/bioinformatics/btq662 PMC3031035

[32] Holm L, Park J (2000) DaliLite workbench for protein structure comparison. Bioinformatics 16: 566–567. 10980157 10.1093/bioinformatics/16.6.566

[33] Horn S (2012) Case study: enrichment of ancient mitochondrial DNA by hybridization capture. Methods Mol Biol 840: 189–195. 22237536 10.1007/978-1-61779-516-9_22

[34] Kircher M (2012) Analysis of high-throughput ancient DNA sequencing data. Methods Mol Biol 840: 197–228. 22237537 10.1007/978-1-61779-516-9_23

[35] Burger J, Hummel S, Hermann B, Henke W (1999) DNA preservation: a microsatellite-DNA study on ancient skeletal remains. Electrophoresis 20: 1722–1728. 10435438 10.1002/(SICI)1522-2683(19990101)20:8<1722::AID-ELPS1722>3.0.CO;2-4

[36] Capelli C, Tschentscher F, Pascali VL (2003) “Ancient” protocols for the crime scene?: Similarities and differences between forensic genetics and ancient DNA analysis. Forensic Sci Int 131: 59–64. 12505472 10.1016/s0379-0738(02)00396-1

[37] Paabo S, Poinar H, Serre D, Jaenicke-Despres V, Hebler J, et al (2004) Genetic analyses from ancient DNA. Annu Rev Genet 38: 645–679. 15568989 10.1146/annurev.genet.37.110801.143214

[38] Bull RA, Eden JS, Luciani F, McElroy K, Rawlinson WD, et al (2012) Contribution of intra- and interhost dynamics to norovirus evolution. J Virol 86: 3219–3229. 22205753 10.1128/JVI.06712-11 PMC3302298

[39] Shojima T, Hoshino S, Abe M, Yasuda J, Shogen H, et al (2013) Construction and characterization of an infectious molecular clone of Koala retrovirus. J Virol 87: 5081–5088. 23427161 10.1128/JVI.01584-12 PMC3624308

[40] Shimode S, Nakagawa S, Yoshikawa R, Shojima T, Miyazawa T (2014) Heterogeneity of koala retrovirus isolates. FEBS Lett 588: 41–46. 24239536 10.1016/j.febslet.2013.10.046

[41] Han JY, Zhao Y, Anderson WF, Cannon PM (1998) Role of variable regions A and B in receptor binding domain of amphotropic murine leukemia virus envelope protein. J Virol 72: 9101–9108. 9765455 10.1128/jvi.72.11.9101-9108.1998 PMC110327

[42] Stoye JP (2001) Endogenous retroviruses: Still active after all these years? Current Biology 11: 914–916. 10.1016/s0960-9822(01)00553-x 11719237

[43] Schuenemann VJ, Bos K, DeWitte S, Schmedes S, Jamieson J, et al (2011) Targeted enrichment of ancient pathogens yielding the pPCP1 plasmid of Yersinia pestis from victims of the Black Death. Proc Natl Acad Sci U S A 108: 746–752. 10.1073/pnas.1105107108 PMC3179067 21876176

[44] Duncavage EJ, Magrini V, Becker N, Armstrong JR, Demeter RT, et al (2011) Hybrid capture and next-generation sequencing identify viral integration sites from formalin-fixed, paraffin-embedded tissue. J Mol Diagn 13: 325–333. 21497292 10.1016/j.jmoldx.2011.01.006 PMC3077736

[45] Yan Y, Buckler-White A, Wollenberg K, Kozak CA (2009) Origin, antiviral function and evidence for positive selection of the gammaretrovirus restriction gene Fv1 in the genus Mus. Proc Natl Acad Sci U S A 106: 3259–3263. 19221034 10.1073/pnas.0900181106 PMC2651326

[46] Hu Y, Tan PT, Tan TW, August JT, Khan AM (2013) Dissecting the dynamics of HIV-1 protein sequence diversity. PLoS One 8(4): e59994. 23593157 10.1371/journal.pone.0059994 PMC3617185

[47] De Feo CJ, Weiss CD (2012) Escape from human immunodeficiency virus type 1 (HIV-1) entry inhibitors. Viruses 4: 3859–3911. 23342377 10.3390/v4123859 PMC3528295

[48] Melder DC, Pankratz VS, Federspiel MJ (2003) Evolutionary pressure of a receptor competitor selects different subgroup a avian leukosis virus escape variants with altered receptor interactions. J Virol 77: 10504–10514. 12970435 10.1128/JVI.77.19.10504-10514.2003 PMC228527

[49] Tsibris AM, Korber B, Arnaout R, Russ C, Lo CC, et al (2009) Quantitative deep sequencing reveals dynamic HIV-1 escape and large population shifts during CCR5 antagonist therapy in vivo. PLoS One 4(5): e5683. 19479085 10.1371/journal.pone.0005683 PMC2682648

[50] Ortiz M, Bleiber G, Martinez R, Kaessmann H, Telenti A (2006) Patterns of evolution of host proteins involved in retroviral pathogenesis. Retrovirology 3: 11. 16460575 10.1186/1742-4690-3-11 PMC1409793

[51] Tarlinton RE, Meers J, Young PR (2006) Retroviral invasion of the koala genome. Nature 442: 79–81. 16823453 10.1038/nature04841

