# Supplementary material for: Hybridization Capture Reveals Evolution and Conservation across the Entire Koala Retrovirus Genome
Source: PLoS One. 2014 Apr 21;9(4):e95633. doi: 10.1371/journal.pone.0095633 (PMC3994108; doi:10.1371/journal.pone.0095633)
Supplement: Figure S1 — Alignment of hybridization capture sequences to koala retrovirus B (KoRV-B) isolate Br2–1CETTG. (PDF) [file pone.0095633.s001.pdf]

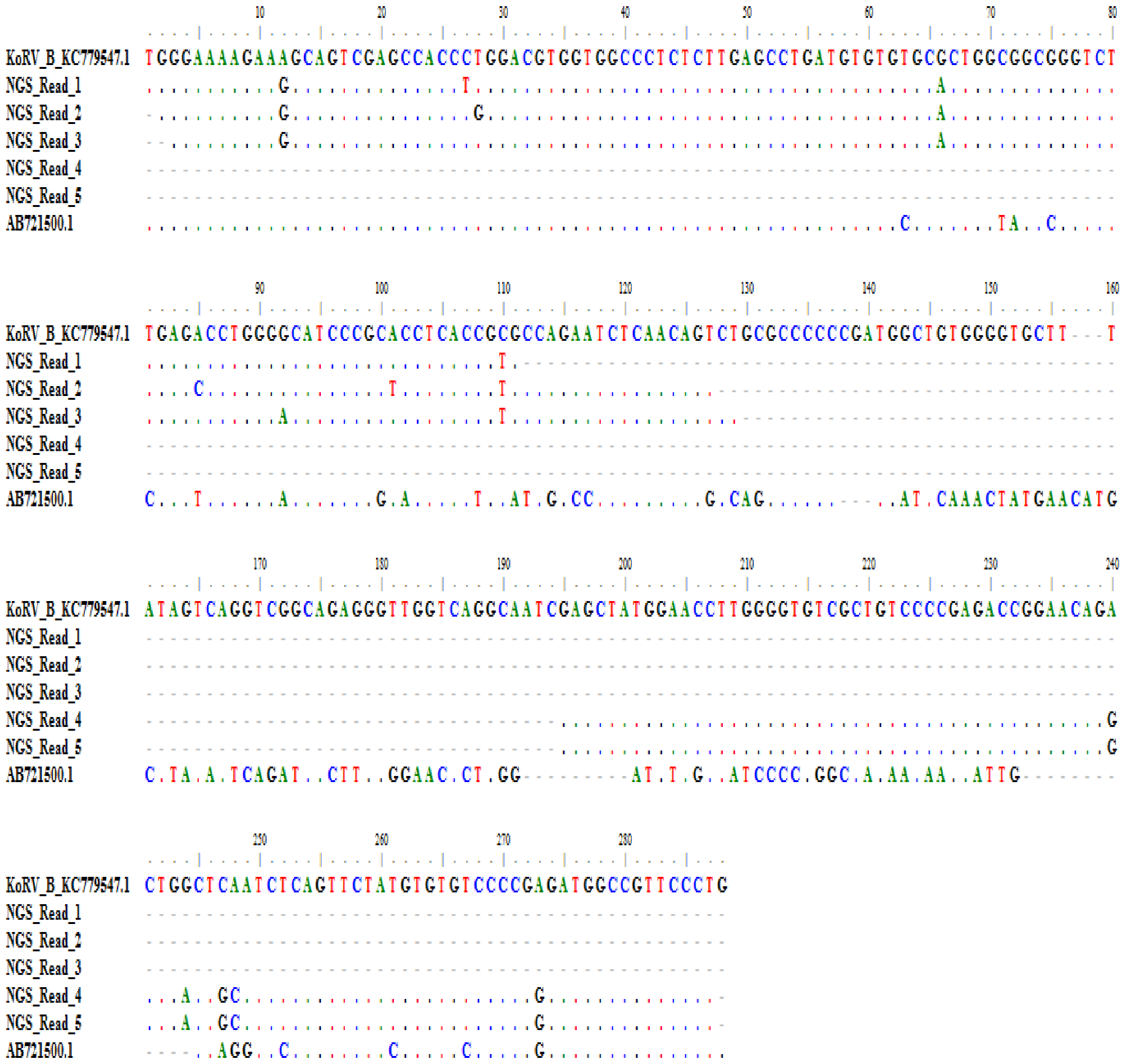

Fig. S1. Alignment of hybridization capture sequences to koala retrovirus B (KoRV-B) isolate Br2-1CETTG. KoRV-B sequences are shown and used as a reference. Individual reads from koala Pci-SN265 were aligned with dots indicating a match, dashes indicating indels, and mismatches indicated by the appropriate DNA base. The KoRV-A sequence AB721500 (Shojima et al. 2013) is included to highlight the differences between KoRV-A and KoRV-B. The sequences shown also correspond to positions 6149-6436 of the reference sequence AF151794 (Hanger et al. 2000).
