# Supplementary material for: Hybridization Capture Reveals Evolution and Conservation across the Entire Koala Retrovirus Genome
Source: PLoS One. 2014 Apr 21;9(4):e95633. doi: 10.1371/journal.pone.0095633 (PMC3994108; doi:10.1371/journal.pone.0095633)
Supplement: Figure S2 — Alignment of hybridization capture sequences to KoRV isolates identified in Japanese zoo koalas: clone 11–1 (panel A) and 11–2 (Panel B). (PDF) [file pone.0095633.s002.pdf]

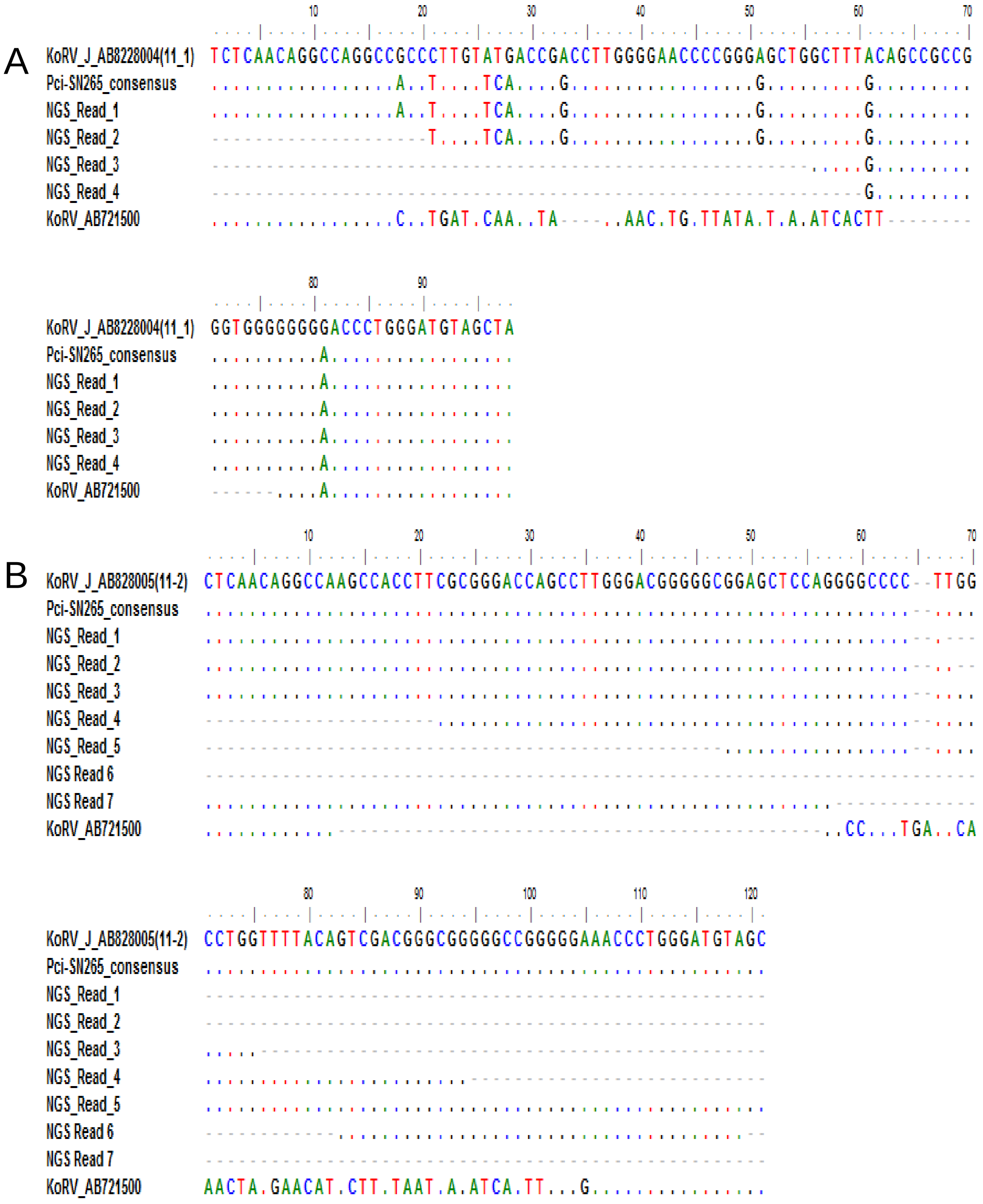

Fig. S2. Alignment of hybridization capture sequences to KoRV isolates identified in Japanese zoo koalas: clone 11-1 (panel A) and 11-2 (Panel B). Clone 11-1 and 11-2 sequences correspond respectively to positions 6205-6303 and 6204-6324 of AF151794 (Hanger et al. 2000). The sequence from each clone is shown as the references to which ale aligned the Pci-SN265 consensus sequence, as well as individual next-generation reads. The sequence of KoRV AB721500 (Shojima et al. 2013) was also aligned to highlight differences versus the two Japanese isolates. Identity to the reference is indicated by a dot, differences by the corresponding base, and indels by a dash.
