# Supplementary material for: Hybridization Capture Reveals Evolution and Conservation across the Entire Koala Retrovirus Genome
Source: PLoS One. 2014 Apr 21;9(4):e95633. doi: 10.1371/journal.pone.0095633 (PMC3994108; doi:10.1371/journal.pone.0095633)
Supplement: Figure S5 — Superimpositions between the modern KoRV (Pci-SN265-green) protein structure and its historical variants show the overall similarity of the structures of the Env protein. (PDF) [file pone.0095633.s005.pdf]

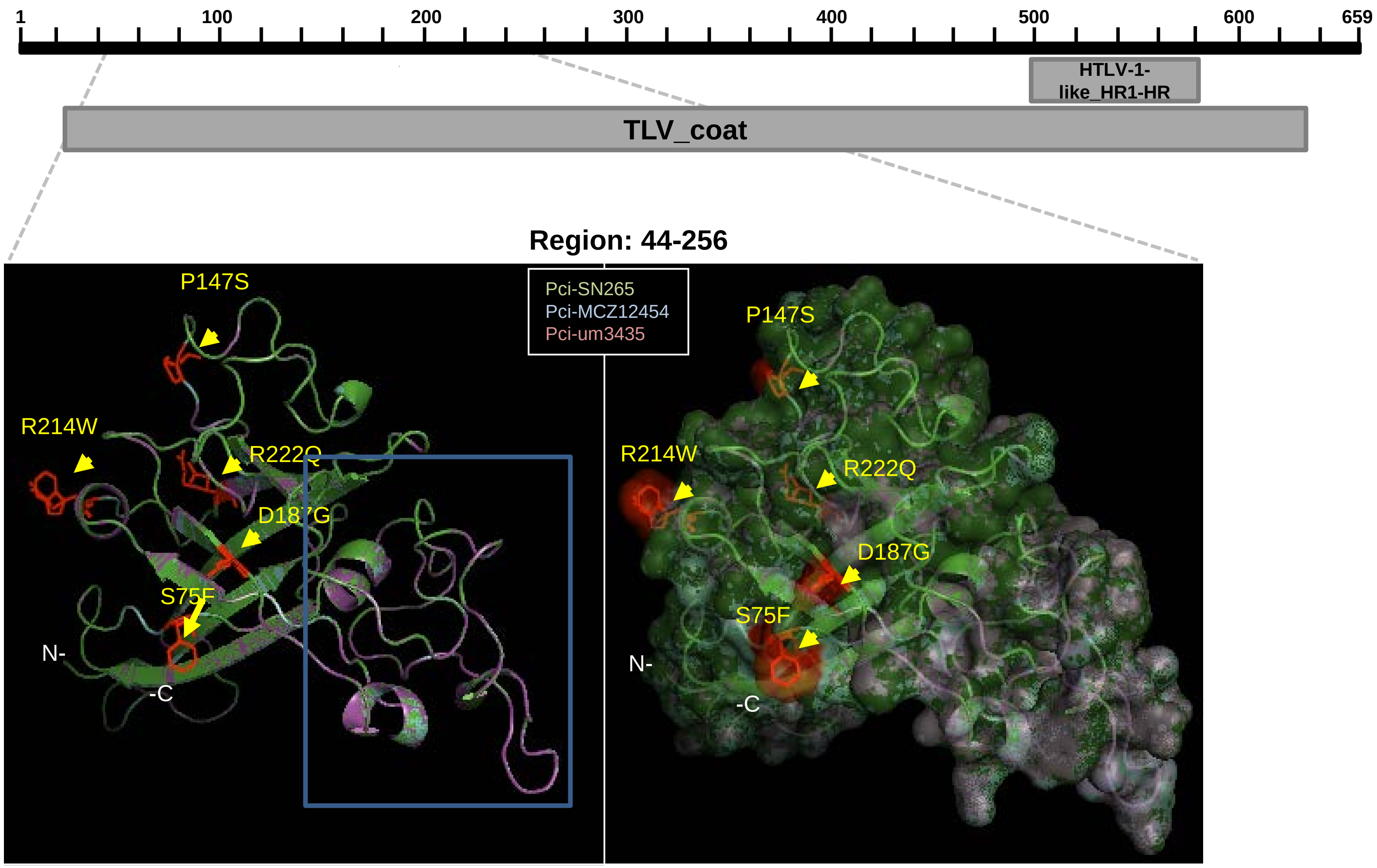

Fig S5. Superimpositions between the modern KoRV (Pci-SN265-green) protein structure and its historical variants show the overall similarity of the structures of the Env protein. Amino acid variations (red) between these sequences are mapped on the protein models (arrows). The structural differences predicted are attributed to changes in the polarity, charge, and atom conformations. These differences are largely localized onto flexible loop regions. The models are shown in cartoon representations and the atoms of the variable amino acid residues in line representations to view the side chains. In all comparisons the Pci-SN265 sequence was used as the reference sequence. The domain organization (as depicted at the CDD database) and the location of the modeled structure of the protein are shown on the top of the figure.
