# Supplementary material for: Hybridization Capture Reveals Evolution and Conservation across the Entire Koala Retrovirus Genome
Source: PLoS One. 2014 Apr 21;9(4):e95633. doi: 10.1371/journal.pone.0095633 (PMC3994108; doi:10.1371/journal.pone.0095633)
Supplement: Table S1 — Koala retrovirus (KoRV) primers. (PDF) [file pone.0095633.s006.pdf]

Table S1: Koala retrovirus (KoRV) primers

Primer Name	Primer Sequence
PCI-KoRV-F1	5'-AGGAGGCAGAAATCATGAGG-3'
PCI-KoRV-R1	5'-AGAAACCCTCCCAGGATCAA-3'
PCI-KoRV-F2	5'-TCGTAAGTTCAATAAACCTCTTGC-3'
PCI-KoRV-R2	5'-ACGTATATTAAAAAGACAGGAAAAAGA-3'
PCI-KoRV-F3	5'-GAGATTCCCACCCAAGGAC-3'
PCI-KoRV-R3	5'-CAGTGATCTAGTGTAAGAGAGAGAGG-3'
PCI-KoRV-F4	5'-CCTGTCTTTTTAATATACGTCTACGC-3'
PCI-KoRV-R4	5'-CGACTTTCGCCCGTTATC-3'
PCI-KoRV-F5	5'-GGGTGAGTCGACCCCTCT-3'
PCI-KoRV-R5	5'-GAGTCCCTCAGCCATTAGGC-3'
PCI-KoRV-F6	5'-AAGATCGCCGTTGCCTCT-3'
PCI-KoRV-R6	5'-AGGAGCTGCTGGCAATCG-3'
PCI-KoRV-F7	5'-ATCCAACGTCCCCTCCAC-3'
PCI-KoRV-R7	5'-GATCCCACTGAGGTCGATT-3'
PCI-KoRV-F8	5'-TTTTCCCACCAGCCTACTTG-3'
PCI-KoRV-R8	5'-AGATCCTGCAAGGAATGGTC-3'
PCI-KoRV-F9	5'-GCCCCTACACAACTCGAGAA-3'
PCI-KoRV-R9	5'-TTTCTCCTGGCGCCTGTC-3'
PCI-KoRV-F10	5'-TTACAAAGGCTGGAAGGACTC-3'
PCI-KoRV-R10	5'-GCTTGGTCAATACTGAATGTTCG-3'
PCI-KoRV-F11	5'-GAGACAGAGGAAAGAGAGAGACG-3'
PCI-KoRV-R11	5'-AAGAATGAGTGGGTCACTTGC-3'
PCI-KoRV-F12	5'-CTGAGTTTTTGGTTGATACCG-3'
PCI-KoRV-R12	5'-TTGCTCATTGGGTACTGTCG-3'
PCI-KoRV-F13	5'-CAAGAGACTTTTGAAAATTGGACA-3'
PCI-KoRV-R13	5'-CGATAGTCATTGGTTCCAGGT-3'
PCI-KoRV-F14	5'-TGAAGTCAGATGCCTCACCA-3'
PCI-KoRV-R14	5'-GTTGAGAGCCCTGAAGGATG-3'
PCI-KoRV-F15	5'-CCTGGAACACCCCTTTGTTA-3'
PCI-KoRV-R15	5'-TTGGCCGACACTCGGTAT-3'
PCI-KoRV-F16	5'-ACTCTCCCACCCTCTTCGAT-3'
PCI-KoRV-R16	5'-GCCTCTTTTATACGGCCAAA-3'
PCI-KoRV-F17	5'-GGGACACGAAGGCTCTTACA-3'
PCI-KoRV-R17	5'-GGCCACCGGATCTAATTTTT-3'
PCI-KoRV-F18	5'-TCCCTTTACCTGGACTGAGG-3'
PCI-KoRV-R18	5'-ATTGGGGTGTCGTCTGACTC-3'
PCI-KoRV-F19	5'-CCCGGTAGCTTACCTGTCAA-3'
PCI-KoRV-R19	5'-GTTCCCTCTGGCAGGTTG-3'
PCI-KoRV-F20	5'-CGGCCATTCTGAATCCTG-3'
PCI-KoRV-R20	5'-CGGGGCAATGGATGATAG-3'
PCI-KoRV-F21	5'-GCCATTGTGGACAACAAGC-3'
PCI-KoRV-R21	5'-GATGCAGCCGTTGAATGAAT-3'
PCI-KoRV-F22	5'-TGCTAGAGGCCATCCATCTC-3'
PCI-KoRV-R22	5'-GCTTGTCTGGCCCTAAGTG-3'
PCI-KoRV-F23	5'-CTACACGGGGGAAGATCAAG-3'
PCI-KoRV-R23	5'-TGTCGGACCCGAGTACCTTA-3'

PCI-KoRV-F24	5'-GAGAGAGCTCACCCCTGACC-3'
PCI-KoRV-R24	5'-CCAATCAATCCCCAGTTGAG-3'
PCI-KoRV-F25	5'-ACTCGGGTCCGACAATGG-3'
PCI-KoRV-R25	5'-CTTTCCACCGAGGCTCAAG-3'
PCI-KoRV-F26	5'-GGTCGGGGTGGTCAGTAG-3'
PCI-KoRV-R26	5'-GGTGGTCAGTAGCACCAGGTA-3'
PCI-KoRV-F27	5'-GTGCTGGTTAGACGTCATCG-3'
KoRVEs-R4.1	5'-GGCCTGGCCTGTTGAGAG-3'
KoRVEs-F26.1	5'-GGAATGCTGTTTCTATGTTGACC-3'
PCI-KoRV-R28	5'-TTTGGCACTCCAAATCTTCC-3'
PCI-KoRV-F29	5'-CAGACCCTAGACAACGAGGA-3'
PCI-KoRV-R29	5'-GAGTACTCGGGCGACTCAG-3'
PCI-KoRV-F30	5'-GTGCCCCTCAGCAGTTTCTA-3'
PCI-KoRV-R30	5'-TGAAAGACCCCAATGTTCG-3'
