# Supplementary material for: Hybridization Capture Reveals Evolution and Conservation across the Entire Koala Retrovirus Genome
Source: PLoS One. 2014 Apr 21;9(4):e95633. doi: 10.1371/journal.pone.0095633 (PMC3994108; doi:10.1371/journal.pone.0095633)
Supplement: Table S2 — KoRV variable sites in modern and historic koalas. (PDF) [file pone.0095633.s007.pdf]

Table S2: KoRV variable sites in modern and historic koalas

Position	20	70	93	111	136	145	147	157	312	357	470	477	492	495	602	604	783	804	806	827
AF151794	A	G	G	G	G	G	G	G	C	T	A	A	T	G	G	C	T	T	C	G
AB721500	A	G	G	G	G	A	A	G	T	C	A	A	T	G	G	C	C	T	T	G
Vienna Zoo	A	G	G/A	G	G	G/A	G/A	G/A	T	C	A	A/T	C/T	G/A	G	C	C/T	C/T	T	G/A
QMJ6480	A	G	G/A	G/A	G	G/A	G/A	G/A	T	C	A	A/T	T	G	G	C	C/T	C/T	T	G/A
521198	A	G	G/A	G	G	G/A	G/A	G	T	C/T	A	A/T	T	G	G	C	C/T	T	C/T	G/A
MCZ12454	A	G	G/A	G	G/A	G/A	G/A	G	T	C/T	A	A/T/G	C/T	G	C/G	C	C/T	T	T	G/A
MCZ8574	A	G	G/A	G	G/A	G/A	G/A	G	T	C	G/A	A/T	T	G	G	C	C/T	T	T/A	G/A
um3435	A	G	G/A	G	G	G/A	G/A	G	T	C/T	A	A/T	T	G	G	C	C/T	T	T	G/A
maex1738	G/A	G/A	G/A	G	G	G/A	G/A	G	T	C	A	A/T	T	G	C/G	C/T	C/T	T	T	G/A
Amino Acid	-	-	-	-	-	-	-	-	-	-	-	-	-	-	-	-	-	-	-	-

Position	866	874	900	909	910	916	964	991	1002	1065	1067	1108	1113	1197	1263	1301	1325	1404	1689	1809
AF151794	G	A	T	C	C	A	C	C	T	G	G	A	G	G	C	G	C	G	T	A
AB721500	G	A	T	C	C	A	A	C	T	G	G	A	G	G	C	G	C	G	C	G
Vienna Zoo	G	A	A/T	C/T	C/T	A	A/C	C	T	G	G	A/G	G	G/A	C/T	G	C	G/A	T/C	A/G
QMJ6480	G	A	T	C/T/G	C/T/G	A	A/C	C	T	G	G	A/G	G/A	G	C	G/A	C	G	T/C	A/G
521198	G	A	T	C/T	C/T	A	A/C	C	T	G	G	A/G	G	G/A	C/T	G	C	G	T/C	A/G
MCZ12454	G	A	T	C/T	C/T	A	A/C	C	T/C	G	G	A/G	G	G	C/T	G	C	G/A	T/C	A/G
MCZ8574	G	A	T	C/T	C/T	A/T	C	C	T	G/A	G	A/G	G	G	C	G	C	G/A	T/C	A/G
um3435	G/T	G/A	T	C/T	C/T	A	A/C	C/T	T	G	G	A/G	G	G	C	G	C	G	T/C	A/G
maex1738	G	A	T	C/T	C/T	A/T	A/C	C	T	G	G/A	A/G	G	G	C	G	C/G	G	T/C	A/G
Amino Acid	-	-	-	-	-	-	-	-	-	-	-	**E/K**	**V**	**P**	**P**	**G/E**	**P/R**	**T**	**L**	**K**

Position	1993	2019	2130	2133	2154	2166	2229	2230	2240	2244	2245	2248	2254	2261	2263	2276	2360	2412	2665	2704
AF151794	G	A	G	G	G	G	T	A	C	G	G	G	C	G	G	C	T	G	T	G
AB721500	G	C	A	G	G	A	T	A	C	G	G	G	C	G	G	C	C	G	C	G
Vienna Zoo	G	A/C	A	G/A	G	G/A	T	A	C	G	G	G	C	G	G	C	C	G	C/T	G/A
QMJ6480	G	A/C	A	G	G	G/A	T	A	C/G	G	G	G	C	G	G	C/T	C	G	C	G/A
521198	G	A/C	A	G	G	G/A	T	A	C/G	G/A	G	G	A/C	G	G	C	C	G	C	G
MCZ12454	G/A	A/C	G/A	G	G/A	G/A	T	A	C/G	G	G/A	G/A	A/C	G	G/A	C	C	G	C	G
MCZ8574	G	A/C	A	G	G	G/A	T	A/G	C/G/A	G	G	G	A/C	G	G	C	C/A	G	C/A	G
um3435	G	A/C	G/A	G	G	G/A	A/T	A	C/G/A	G	G/A	G/A	A/C	G	G	C	C	G	C/T	G
maex1738	G	A/C	A	G	G	G/A	T	A/G	C/G	G	G	G	A/C	G/A	G/A	C	C/T	G/A	C	G/A
Amino Acid	**V/I**	**T**	**G**	**Q**	**K**	**R**	**H/Q**	**K/E**	**T/R/K**	**E**	**E/K**	**E/K**	**Q/K**	**K/R**	**E/K**	**T/I**	**S/T/Y**	**R**	**S/T/P**	**V/I**

Position	2880	2922	2926	3020	3069	3235	3306	3336	3572	3672	3717	3719	3736	3810	3836	4212	4485	4675	4702	4725
AF151794	C	A	G	T	T	G	G	C	C	C	G	G	C	A	T	C	A	T	G	A
AB721500	C	A	G	T	T	G	G	C	C	C	G	G	C	A	T	T	A	T	G	G
Vienna Zoo	C/T	G/A	G	T/C	T	G	G	C/T	C	C	G	G	C	G/A	T	T	A/G	T	G	A/G
QMJ6480	C/T	G/A	G	T/C	T	G	G	C	C	C	G	G	C	A	T	T/A	A/G	T	G	A/G
521198	C/T	G/A	G	T/C	T	G	G	C	C/A	C	G	G	C	A	T	T	A/G	T	G	A/G
MCZ12454	C/T	A	G	T/C	T/C	G/A	G/T	C	C	C	G	G	C	A	T	T	A	T	G	A/G
MCZ8574	C	G/A	G	T/C	T	G	G	C	C	C	G	G	C/T	A	T	T/C	A	T	G/T	A/G
um3435	C/T	G/A	G	T/C	T	G	G	C/T	C	C/T	G	G	C	A	T	T/A	A/G	T/A	G	A/G
maex1738	C/T	G/A	G/A	T/C	T	G	G/T	C	C	C	G/A	G/A	C	A	T/A	T/C	A/G	T	G	A/G
Amino Acid	**P**	**L**	**E/K**	**A/V**	**V**	**E/K**	**P**	**D**	**A**	**C**	**K**	**R**	**R/***	**G**	**F/Y**	**S/R**	**R**	**Y**	**A/S**	**K**

Position	4940	4960	5004	5113	5133	5134	5179	5207	5227	5419	5446	5465	5639	5646	5690	6138	6157	6353	6474	6541
AF151794	G	G	G	G	G	C	G	G	C	G	C	T	C	G	C	C	C	T	G	C
AB721500	G	G	A	A	A	T	G	G	C	A	C	T	C	G	C	C	C	T	G	C
Vienna Zoo	G/A	G/A	G/A	G/A	G/A	T	G	G	C	G/A	C	T	C/T	G/A	C	C	C/T	T/C	G/A	C
QMJ6480	G/A	G/A	G/A	G/A	G/A	C/T	G/A	G	C	G/A	C	T	C/T	G	C	C	C/T	T	G/A	C/T
521198	G/A	G/A	G/A	G/A	G/A	C/T	G	G	C	G/A	C	T	C	G/A	C	C	C/T	T	G	C
MCZ12454	G/A	G/A	A	G/A	G/A	T/G	G	G	C	G/A	C	T	C	G	C/T	C/T	C/T	T	G/A	C
MCZ8574	G/A	G/A	A	G/A	G/A	T/G	G	G	C	G/A	C	T	C	G	C	C	C/T	T	G	C
um3435	G/A	G/A	G/A	G/A	G/A	C/T	G	G/A	C/A	G/A	C	T/A	C	G	C	C	C/T	T	G	C
maex1738	G/A	G/A	G/A	G/A	G/A	T/A	G	G	C	G/A	C/A	T	C	G	C	C	C/T	T	G	C
Amino Acid	**R/K**	**R/G**	**G**	**A/T**	**E**	**P/S**	**V/I**	**R/Q**	**L/I**	**N/D**	**P/T**	**V/E**	**A/V**	**G**	**T/I**	**S/F**	**C**	**S/P**	**G/D**	**T**

Position	6554	6579	6710	6717	6765	6779	6922	6964	6993	7046	7097	7137	7140	7287	7289	7336	7381	7891	7925	7933
AF151794	C	G	A	C	C	C	T	T	G	G	A	G	G	A	T	C	A	T	G	G
AB721500	C	G	A	C	C	C	T	T	G	G	A	A	G	A	C	C	A	T	G	G
Vienna Zoo	C	G	A/G	C	C/T	C	T/C	T	G	G	A	A	G	A	C	C	A/G	T/C	G	G
QMJ6480	C	G	A/G	C/T	C	C	T/C	T	G/A	G	A	A	G/A	A	T/C	C	A	T/C	G	G
521198	C	G	A/G	C	C/T	C	T/C	T/A	G	G	A	A	G	A	T/C/A	C	A	T/C	G/A	G/A
MCZ12454	C/T	G	A/G	C	C	C	T/C	T	G/A	G	A	A	G	A/T	C	C/T	A	T/C	G	G
MCZ8574	C	G	A/G	C	C/T	C/T	T/C	T	G	G/A	A/G	A/C	G	A	C	C	A	T/C	G/A	G/A
um3435	C	G/A	A/G	C	C	C	T/C	T/A	G	G	A	A/C	G	A	T/C	C	A	T/C	G	G
maex1738	C	G	A/G	C	C	C	T/C	T/A	G/A	G	A	A/T	G	A/T	T/C	C	A	T/C	G	G
Amino Acid	**R/W**	**R/Q**	**K/E**	**P/L**	**P/L**	**Q/***	**P**	**Y/***	**G/E**	**G/R**	**N/D**	**R/H/P/K**	**S/N**	**H/P**	**P/S**	**L**	**L**	**L**	-	-

Position	7938	8071	8076	8080	8091	8092	8405	8410	8412	8426	8430	8439	8440
AF151794	T	G	A	G	G	G	T	A	A	A	G	A	T
AB721500	T	G	G	A	G	G	T	G	A	A	G	A	T
Vienna Zoo	T	G/A	A/G	G/A	G/A	G	T/C	A/G	A/T	A/T	G/A	A	T/A
QMJ6480	T	G	A/G	G/A	G	G/A	T/C	G	A/T	A	G	A	T/G
521198	T	G	A/G	G/A	G	G	T/C	A/G	A/T	A	G	A	T/A
MCZ12454	T	G	A/G	G/A	G	G	T/C	A/G	A/T	A	G	A	T/C
MCZ8574	T	G	A/G	G/A	G	G	T/C	A/G	A/T	A	G	A	T
um3435	T	G	A/G	G/A	G	G	T/C	G	A/T	A	G	A	T/A
maex1738	T/G	G	A/G	G/A	G	G	T/C	G	A/T	A	G	A/T	G
Amino Acid	-	-	-	-	-	-	-	-	-	-	-	-	-

-Non coding regions

Position numbers follow those of the KoRV reference genome, GenBank accession AF151794

The first two sequences are from Genbank; the "Vienna Zoo" koala is PCI-SN265; for other koala specimens see Table 1

Variation present in more than two koalas (shared)

Variation present in one koala (private)

Fixed difference

The first amino acid corresponds to AF151794; the second or subsequent amino acids are in order of appearance in the rows. Asterisk (*) indicates a stop codon
