# Supplementary material for: Hybridization Capture Reveals Evolution and Conservation across the Entire Koala Retrovirus Genome
Source: PLoS One. 2014 Apr 21;9(4):e95633. doi: 10.1371/journal.pone.0095633 (PMC3994108; doi:10.1371/journal.pone.0095633)
Supplement: Table S3 — Estimates of the overall dn and ds distance and the dn/ds ratios. (PDF) [file pone.0095633.s008.pdf]

Table S3. Estimates of the overall *dn and ds* distance and the dn/ds ratios

	dn	*ds*	*dn/ds*
gag	0.003±0.001	0.013±0.003	0,23
pol	0.002±0.001	0.005±0.002	0,4
env	0.003±0.001	0.004±0.002	0,75

*To include the polymorphisms in all phases the analysis involved 16 KoRV sequences.
Codon positions included were 1st+2nd+3rd.
All positions containing gaps or missing data were eliminated.
There were 521 codon positions for gag, 1126 for pol, and 657 for env in the final dataset.
