# Supplementary material for: Hybridization Capture Reveals Evolution and Conservation across the Entire Koala Retrovirus Genome
Source: PLoS One. 2014 Apr 21;9(4):e95633. doi: 10.1371/journal.pone.0095633 (PMC3994108; doi:10.1371/journal.pone.0095633)
Supplement: Table S4 — Estimates of Tajima’s D. (PDF) [file pone.0095633.s009.pdf]

Table S4. Estimates of Tajima's D*

	m	S	ps	Θ	π	D
gag	16	34	0,02175	0,00656	0,00545	-0,699737
pol	16	37	0,01095	0,0033	0,0031	-0,253388
env	16	22	0,01116	0,00336	0,00304	-0,390221

*All positions containing gaps or missing data were eliminated. There were 1563 positions for gag, 3378 for pol, and 1971 for env in the final dataset.
Abbreviations: m = number of sequences; S = Number of segregating sites; ps = S/m; Θ = ps/a1; π = nucleotide diversity
D is the Tajima test statistic
