# Supplementary material for: Hybridization Capture Reveals Evolution and Conservation across the Entire Koala Retrovirus Genome
Source: PLoS One. 2014 Apr 21;9(4):e95633. doi: 10.1371/journal.pone.0095633 (PMC3994108; doi:10.1371/journal.pone.0095633)
Supplement: Table S5 — Codon based Z tests of selection. (PDF) [file pone.0095633.s010.pdf]

Table S5. Codon based Z tests of selection

	1	2	3	4	5	6	7	8	9	10	11	12	13	14	15	16
gag (521 codons)																
Pci_SN265		0	0	-1,049	-0,65	-0,662	-1,015	-0,677	-1,817	-2,716	-1,775	-2,011	-2,221	-2,138	-0,794	-1,318
Pci_QMJ6480	1		0	-1,049	-0,65	-0,662	-1,015	-0,677	-1,817	-2,716	-1,775	-2,011	-2,221	-2,138	-0,794	-1,318
Pci_582119	1	1		-1,049	-0,65	-0,662	-1,015	-0,677	-1,817	-2,716	-1,775	-2,011	-2,221	-2,138	-0,794	-1,318
Pci_MCZ_12454	0,296	0,296	0,296		-1,17	0,988	-1,499	1,017	-2,068	-2,902	-2,002	-2,24	-2,425	-2,327	-1,14	-1,584
Pci_MCZ8574	0,517	0,517	0,517	0,244		-1,456	-1,145	-0,939	-1,856	-2,751	-1,998	-2,219	-2,411	-2,086	-1,269	-1,577
Pci_um3435	0,509	0,509	0,509	0,325	0,148		-1,218	1,441	-1,886	-2,749	-2,004	-2,242	-2,427	-2,484	-1,298	-1,586
Pci_maex1738	0,312	0,312	0,312	0,137	0,254	0,225		-1,225	-2,099	-2,914	-2,023	-2,249	-2,464	-2,373	-1,153	-1,003
1Pci_SN265	0,5	0,5	0,5	0,311	0,35	0,152	0,223		-1,91	-2,787	-1,88	-2,106	-2,315	-2,211	-1,001	-1,462
1Pci_QMJ6480	0,072	0,072	0,072	0,041	0,066	0,062	0,038	0,059		-2,09	-0,682	-0,948	-1,331	-1,154	2,63	0,173
1Pci_582119	0,008	0,008	0,008	0,004	0,007	0,007	0,004	0,006	0,039		-2,026	-0,913	-1,306	-1,793	-0,989	-1,489
1Pci_MCZ_12454	0,078	0,078	0,078	0,048	0,048	0,047	0,045	0,063	0,496	0,045		-1,624	-1,837	-1,55	0,421	-0,688
1Pci_MCZ8574	0,047	0,047	0,047	0,027	0,028	0,027	0,026	0,037	0,345	0,363	0,107		-1,565	-1,94	-0,249	-1,165
1Pci_um3435	0,028	0,028	0,028	0,017	0,017	0,017	0,015	0,022	0,186	0,194	0,069	0,12		-1,655	-1,46	-1,728
1Pci_maex1738	0,035	0,035	0,035	0,022	0,039	0,014	0,019	0,029	0,251	0,076	0,124	0,055	0,1		-0,766	-1,488
KoRV_AF151794.2_Hanger	0,429	0,429	0,429	0,256	0,207	0,197	0,251	0,319	0,01	0,324	0,675	0,804	0,147	0,445		0,74
KoRV_AB721500	0,19	0,19	0,19	0,116	0,117	0,115	0,318	0,146	0,863	0,139	0,493	0,246	0,087	0,139	0,461	
pol (1126 codons)																
Pci_SN265		-0,293	-0,293	-0,618	-0,618	-1,161	-0,618	-1,175	-1,461	-1,828	-1,422	-1,924	-1,202	-0,277	-1,906	-1,681
Pci_QMJ6480	0,77		0	1,023	1,47	-0,614	1,023	-0,657	-1,137	-2,017	-1,146	-1,707	-0,989	0,013	-1,66	-1,431
Pci_582119	0,77	1		1,023	1,47	-0,614	1,023	-0,657	-1,137	-2,017	-1,146	-1,707	-0,989	0,013	-1,66	-1,431
Pci_MCZ_12454	0,538	0,308	0,308		1,03	-0,283	0	-0,308	-1,344	-2,142	-1,294	-1,85	-0,989	0,013	-1,791	-1,564
Pci_MCZ8574	0,538	0,144	0,144	0,305		-0,283	1,03	-0,308	-1,13	-2,007	-1,153	-1,712	-0,847	0,18	-1,671	-1,444
Pci_um3435	0,248	0,541	0,541	0,778	0,778		-0,283	-1,342	-1,322	-1,886	-1,009	-1,573	-0,847	-0,285	-1,542	-1,314
Pci_maex1738	0,538	0,308	0,308	1	0,305	0,778		-0,308	-1,344	-2,142	-1,294	-1,85	-0,989	0,013	-1,791	-1,564
1Pci_SN265	0,242	0,513	0,513	0,759	0,759	0,182	0,759		-1,323	-2,092	-1,284	-1,816	-1,18	-0,292	-1,772	-1,548
1Pci_QMJ6480	0,147	0,258	0,258	0,182	0,261	0,189	0,182	0,188		-1,68	-0,514	-1,269	-0,977	0,193	-1,239	-0,95
1Pci_582119	0,07	0,046	0,046	0,034	0,047	0,062	0,034	0,039	0,096		-1,473	-0,635	-2,112	-1,556	-0,715	-1,35
1Pci_MCZ_12454	0,158	0,254	0,254	0,198	0,251	0,315	0,198	0,202	0,608	0,143		0,286	-1,232	-0,333	0,009	0,011
1Pci_MCZ8574	0,057	0,09	0,09	0,067	0,089	0,118	0,067	0,072	0,207	0,527	0,775		-1,841	-1,126	-0,962	-0,496
1Pci_um3435	0,232	0,324	0,324	0,324	0,399	0,399	0,324	0,24	0,331	0,037	0,22	0,068		-1,132	-1,822	-0,937
1Pci_maex1738	0,782	0,989	0,989	0,989	0,858	0,776	0,989	0,771	0,848	0,122	0,74	0,263	0,26		-1,121	-0,818
KoRV_AF151794.2_Hanger	0,059	0,1	0,1	0,076	0,097	0,126	0,076	0,079	0,218	0,476	0,993	0,338	0,071	0,264		-0,607
KoRV_AB721500	0,095	0,155	0,155	0,12	0,151	0,191	0,12	0,124	0,344	0,18	0,992	0,621	0,351	0,415	0,545	
env (657 codons)																
Pci_SN265		-0,202	0,012	0,012	0,583	0,584	0,018	-1,29	-1,108	-1,108	-1,102	-1,29	-1,108	-1,436	-0,923	-1,29
Pci_QMJ6480	0,84		0,834	-0,2	1,354	0,861	-0,199	-1,089	-0,912	-0,912	-0,913	-1,089	-0,912	-1,24	-0,743	-1,089
Pci_582119	0,991	0,406		0,992	2,165	2,142	0,582	-1,12	-1,12	-1,12	-0,905	-1,12	-1,12	-1,477	-0,899	-1,12
Pci_MCZ_12454	0,991	0,841	0,323		1,434	1,011	-0,422	-0,9	-0,737	-0,737	-1,068	-0,9	-0,737	-1,089	-0,573	-0,9
Pci_MCZ8574	0,561	0,178	0,032	0,154		2,052	0,565	-0,698	-0,511	-0,511	-0,509	-0,698	-0,511	-0,919	-0,51	-0,698
Pci_um3435	0,56	0,391	0,034	0,314	0,042		0,013	-1,091	-0,888	-0,888	-0,886	-1,091	-0,888	-1,266	-0,887	-1,091
Pci_maex1738	0,986	0,843	0,562	0,674	0,573	0,99		-1,273	-1,099	-1,099	-1,085	-1,273	-1,099	-0,699	-1,098	-1,273
1Pci_SN265	0,199	0,278	0,265	0,37	0,486	0,277	0,205		1,02	1,02	1,036	0	1,02	-0,664	1,449	0
1Pci_QMJ6480	0,27	0,364	0,265	0,463	0,61	0,376	0,274	0,31		0	1,38	1,02	0	-1,045	1,067	1,02
1Pci_582119	0,27	0,364	0,265	0,463	0,61	0,376	0,274	0,31	1		1,38	1,02	0	-1,045	1,067	1,02
1Pci_MCZ_12454	0,272	0,363	0,367	0,288	0,612	0,378	0,28	0,302	0,17	0,17		1,036	1,38	-0,3	1,723	1,036
1Pci_MCZ8574	0,199	0,278	0,265	0,37	0,486	0,277	0,205	1	0,31	0,31	0,302		1,02	-0,664	1,449	0
1Pci_um3435	0,27	0,364	0,265	0,463	0,61	0,376	0,274	0,31	1	1	0,17	0,31		-1,045	1,067	1,02
1Pci_maex1738	0,154	0,217	0,142	0,278	0,36	0,208	0,486	0,508	0,298	0,298	0,765	0,508	0,298		-0,651	-0,664
KoRV_AF151794.2_Hanger	0,358	0,459	0,37	0,568	0,611	0,377	0,275	0,15	0,288	0,288	0,088	0,15	0,288	0,516		1,449
KoRV_AB721500	0,199	0,278	0,265	0,37	0,486	0,277	0,205	1	0,31	0,31	0,302	1	0,31	0,508	0,15	

The test statistic dN-dS is shown above the diagonal. dN and dS are the values of non-synonymous and synonymous substitutions per site, respectively. The Nei-Gojobori method was used to calculate synonymous and nonsynonymous substitutions.
The probability of rejecting the null hypothesis of strict-neutrality (dN = dS) is shown below the diagonal. Values of P less than 0.05 are highlighted in gray. The variance of the difference was computed using the bootstrap method (500 replicates). Numbers listed for columns represent the same KoRV sequences numbered in the rows. The last two KoRV sequences are from GenBank; the other KoRVs are sequences from the current study.
