# Supplementary material for: Hybridization Capture Reveals Evolution and Conservation across the Entire Koala Retrovirus Genome
Source: PLoS One. 2014 Apr 21;9(4):e95633. doi: 10.1371/journal.pone.0095633 (PMC3994108; doi:10.1371/journal.pone.0095633)
Supplement: Table S6 — Variation in putative transcription factor binding sites in KoRV LTRs. (PDF) [file pone.0095633.s011.pdf]

**Table S6: Variation in putative transcription factor binding sites in KoRV LTRs**

| | Samples | | | | | | | | |
|---|---|---|---|---|---|---|---|---|---|
| Family | AF151794.2 | AB721500.1 | Pci-SN265 | Pci-QMJ648( | Pci-582119 | Pci-MCZ12454 | Pci-MCZ8574 | Pci-um3435 | Pci-maex1738 |
| Core promoter initiator elements | 1 | 1 | 1 | 1 | 1 | 1 | 1 | 1 | 1 |
| Vertebrate TATA binding protein factor | 1 | 1 | 1 | 1 | 1 | 1 | 1 | 1 | 1 |
| Abdominal-B type homeodomain transcription factors | 3 | 3 | 3 | 3 | 3 | 3 | 3 | 3 | 3 |
| MAF and AP1 related factors | 1 | 1 | 1 | 1 | 1 | 1 | 1 | 1 | 1 |
| Bicoid-like homeodomain transcription factors | 1 | 1 | 1 | 1 | 1 | 1 | 1 | 1 | 1 |
| POZ domain zinc finger expressed in B-Cells | 3 | 3 | 3 | 3 | 3 | 3 | 3 | 3 | 3 |
| Brn-5 POU domain factors | 2 | 2 | 2 | 2 | 2 | 2 | 2 | 2 | 2 |
| CCAAT binding factors | 1 | 2 | 1 | 2 | 1 | 1 | 2 | 1 | 1 |
| Calcium-response elements | 1 | 1 | 1 | 1 | 1 | 1 | 1 | 1 | 1 |
| Cell cycle regulators | 1 | 1 | 1 | 1 | 1 | 1 | 1 | 1 | 1 |
| Vertebrate caudal related homeodomain protein | 2 | 2 | 2 | 2 | 2 | 2 | 2 | 2 | 2 |
| Ccaat/Enhancer Binding Protein | 2 | 2 | 2 | 3 | 2 | 2 | 2 | 2 | 2 |
| Cell cycle regulators: Cell cycle homology element | 1 | 1 | 1 | 1 | 1 | 1 | 1 | 1 | 1 |
| CLOX and CLOX homology (CDP) factors | 1 | 1 | 1 | 1 | 1 | 1 | 1 | 1 | 1 |
| CP2-erythrocyte Factor related to drosophila Elf1 | 0 | 0 | 1 | 0 | 0 | 0 | 1 | 0 | 0 |
| CTCF and BORIS gene family | 1 | 1 | 1 | 1 | 1 | 1 | 1 | 1 | 1 |
| Cyclin D binding myb-like transcription factor | 1 | 1 | 1 | 1 | 1 | 1 | 1 | 1 | 1 |
| E2F-myc activator/cell cycle regulator | 4 | 4 | 4 | 4 | 4 | 4 | 4 | 4 | 4 |
| E-box binding factors | 2 | 2 | 2 | 2 | 2 | 2 | 2 | 2 | 2 |
| Estrogen response elements | 2 | 2 | 2 | 2 | 2 | 2 | 2 | 2 | 2 |
| Human and murine ETS1 factors | 6 | 5 | 5 | 5 | 5 | 5 | 5 | 5 | 5 |
| EVI1-myleoid transforming protein | 0 | 0 | 0 | 1 | 0 | 0 | 0 | 0 | 1 |
| FAST-1 SMAD interacting proteins | 1 | 1 | 1 | 1 | 1 | 1 | 1 | 1 | 1 |
| Fork head domain factors | 4 | 5 | 4 | 5 | 4 | 4 | 5 | 4 | 4 |
| Farnesoid X - activated receptor response elements | 1 | 1 | 1 | 1 | 1 | 1 | 1 | 1 | 1 |
| Growth factor independence transcriptional repressor | 1 | 1 | 1 | 1 | 1 | 1 | 1 | 1 | 1 |
| Glucocorticoid responsive and related element | 1 | 1 | 2 | 2 | 1 | 1 | 1 | 1 | 1 |
| Human acute myelogenous leukemia factors | 1 | 1 | 1 | 1 | 1 | 1 | 1 | 1 | 1 |
| Twist subfamily of class B bHLH transcription factors | 3 | 2 | 2 | 2 | 2 | 2 | 3 | 2 | 2 |
| Heat shock factors | 5 | 5 | 5 | 5 | 5 | 5 | 5 | 5 | 5 |
| Vertebrate homologues of enhancer of split complex | 3 | 3 | 3 | 3 | 3 | 3 | 3 | 3 | 3 |
| Krueppel-like C2H2 zinc finger factors hypermethylated in cancer | 1 | 1 | 1 | 1 | 1 | 1 | 1 | 1 | 1 |
| Human muscle-specific Mt binding site | 1 | 1 | 1 | 1 | 1 | 1 | 1 | 1 | 1 |
| Onecut homeodomain factor HNF6 | 1 | 1 | 1 | 1 | 1 | 1 | 1 | 1 | 1 |
| HOX - PBX complexes | 2 | 2 | 2 | 2 | 2 | 2 | 2 | 2 | 2 |
| HOX - MEIS1 heterodimers | 0 | 0 | 0 | 0 | 0 | 0 | 0 | 0 | 1 |
| Ikaros zinc finger family | 1 | 1 | 1 | 1 | 1 | 1 | 1 | 1 | 1 |
| Interferon regulatory factors | 1 | 1 | 1 | 1 | 1 | 1 | 1 | 1 | 1 |
| Krueppel like transcription factors | 2 | 2 | 2 | 2 | 2 | 2 | 2 | 2 | 2 |
| LEF1/TCF | 2 | 2 | 2 | 2 | 2 | 2 | 2 | 2 | 2 |
| Myc associated zinc fingers | 1 | 1 | 1 | 1 | 1 | 1 | 1 | 1 | 1 |
| MEF3 binding sites | 1 | 1 | 1 | 1 | 1 | 1 | 1 | 1 | 1 |
| Myc-interacting Zn finger protein 1 | 1 | 1 | 1 | 1 | 1 | 1 | 1 | 1 | 1 |
| Cellular and viral myb-like transcriptional regulators | 3 | 2 | 3 | 2 | 2 | 3 | 2 | 2 | 2 |
| Myoblast determining factors | 1 | 1 | 1 | 1 | 1 | 1 | 1 | 1 | 1 |
| MYT1 C2HC zinc finger protein | 0 | 0 | 0 | 0 | 0 | 0 | 0 | 0 | 1 |
| Myeloid zinc finger 1 factors | 1 | 1 | 1 | 1 | 1 | 1 | 1 | 1 | 1 |
| NGFI-B response elements, nur subfamily of nuclear receptors | 1 | 1 | 1 | 1 | 1 | 1 | 1 | 1 | 1 |
| Nuclear factor 1 | 0 | 1 | 1 | 1 | 1 | 1 | 1 | 1 | 1 |
| Nuclear factor of activated T-cells | 1 | 1 | 1 | 1 | 1 | 1 | 1 | 1 | 1 |
| Nuclear factor kappa B/c-rel | 1 | 1 | 1 | 1 | 1 | 1 | 1 | 1 | 1 |
| Octamer binding protein | 5 | 5 | 5 | 6 | 5 | 5 | 5 | 5 | 1 |
| Odd-skipped related factors | 0 | 1 | 1 | 1 | 1 | 1 | 1 | 1 | 1 |
| OVO homolog-like transcription factors | 1 | 1 | 1 | 1 | 1 | 1 | 1 | 1 | 5 |
| PAR/bZIP family | 3 | 3 | 3 | 4 | 3 | 3 | 3 | 3 | 3 |
| PAX-4/PAX-6 paired domain binding sites | 1 | 1 | 1 | 1 | 1 | 1 | 1 | 1 | 3 |
| Peroxisome proliferator-activated receptor | 1 | 1 | 1 | 1 | 1 | 1 | 1 | 1 | 1 |
| Pleomorphic adenoma gene | 3 | 3 | 3 | 3 | 3 | 3 | 3 | 3 | 1 |
| v-ERB and RAR-related orphan receptor alpha | 1 | 1 | 1 | 1 | 1 | 1 | 1 | 1 | 2 |
| SWI/SNF related nucleophosphoproteins with a RING finger DNA binding motif | 1 | 1 | 1 | 1 | 1 | 1 | 1 | 1 | 3 |
| RXR heterodimer binding sites | 2 | 2 | 2 | 2 | 2 | 2 | 2 | 2 | 2 |
| Spalt-like transcription factor 2 | 1 | 1 | 1 | 1 | 1 | 1 | 1 | 1 | 1 |
| Vertebrate steroidogenic factor | 1 | 1 | 1 | 1 | 1 | 1 | 1 | 1 | 3 |
| Sine oculis (SIX) homeodomain factors | 1 | 1 | 1 | 1 | 1 | 1 | 1 | 1 | 1 |
| Vertebrate SMAD family of transcription factors | 0 | 0 | 1 | 0 | 0 | 0 | 1 | 0 | 1 |
| SOX/SRY-sex/testis determinig and related HMG box factors | 2 | 2 | 2 | 2 | 2 | 2 | 2 | 2 | 2 |
| GC-Box factors SP1/GC | 1 | 1 | 1 | 1 | 1 | 1 | 1 | 1 | 1 |
| Testis-specific bHLH-Zip transcription factors | 1 | 1 | 1 | 1 | 1 | 1 | 1 | 1 | 2 |
| Signal transducer and activator of transcription | 2 | 2 | 2 | 2 | 2 | 2 | 2 | 2 | 1 |
| Motif composed of binding sites for pluripotency or stem cell factors | 3 | 3 | 4 | 3 | 3 | 3 | 3 | 3 | 1 |
| TEA/ATTS DNA binding domain factors | 1 | 1 | 1 | 1 | 1 | 1 | 1 | 1 | 2 |
| X-box binding factors | 1 | 1 | 1 | 1 | 1 | 1 | 1 | 1 | 3 |
| Y-box binding transcription factors, | 1 | 1 | 1 | 1 | 1 | 1 | 1 | 1 | 1 |
| C2H2 zinc finger transcription factors 2 | 1 | 1 | 1 | 1 | 1 | 1 | 1 | 1 | 1 |
| C2H2 zinc finger transcription factors 3 | 0 | 0 | 1 | 0 | 0 | 1 | 0 | 0 | 0 |
| C2H2 zinc finger transcription factors 10 | 1 | 1 | 1 | 1 | 1 | 1 | 1 | 1 | 1 |
| ZF5 POZ domain zinc finger | 0 | 1 | 1 | 1 | 0 | 1 | 0 | 0 | 1 |
| Two-handed zinc finger homeodomain transcription factors | 2 | 2 | 2 | 2 | 2 | 2 | 2 | 2 | 2 |
| Retroviral CCAAT binding factors | 2 | 2 | 2 | 2 | 2 | 2 | 2 | 2 | 2 |
| Retroviral PolyA Downstream signal | 1 | 1 | 1 | 1 | 1 | 1 | 1 | 1 | 1 |
| Retroviral PolyA signal | 1 | 1 | 1 | 1 | 1 | 1 | 1 | 1 | 1 |
| Retroviral upstream element | 2 | 2 | 2 | 2 | 2 | 2 | 2 | 2 | 2 |

Grey shading indicates sequences with differing numbers of predicted TFBs for a given domain
