# Supplementary material for: Hybridization Capture Reveals Evolution and Conservation across the Entire Koala Retrovirus Genome
Source: PLoS One. 2014 Apr 21;9(4):e95633. doi: 10.1371/journal.pone.0095633 (PMC3994108; doi:10.1371/journal.pone.0095633)
Supplement: Table S7 — KoRV flanking sequences common to two or more koalas. (PDF) [file pone.0095633.s012.pdf]

Table S7: KoRV flanking sequences common to two or more koalas

	Sequence	Total	Pci-SN265	Pci-QMJ6480	Pci-582119	Pci-MCZ12454	Pci-MCZ8574	Pci-um3435	Pci-maex1738	Pci-SN404(WG (1/16))	Pci-SN404	Pci-SN248	Pci-SN345	Pci-157	Pci-106	Pci-182
' flanking sequences																
PCI -5'Flank-1	5'-GAAGGAAGGAAGGAAGGAAGGAAGGAA-3'	5		+		+	+		+	+						
PCI -5'Flank-2	5'-GTTCCCTGTCTTTACAAACTGYAAAAGAAAGAAAACGAGGAA-3'	3	+	+				+								
PCI -5'Flank-3	5'-CTGCAAAAGAAAGAAAATGGGGGAA-3'	2						+	+							
PCI -5'Flank-4	5'-AAGAAAGAAAGAAAGAAAGAAAGAAAGAAAATGGGGGAA-3'	2				+		+								
PCI -5'Flank-5	5'-ATTCAGAAGAAAAATGGGGGAA-3'	2			+		+									
PCI -5'Flank-6	5'-AGCAATGMAARGARAKASAAGAAAATGGGGGAA-3'	2					+	+								
PCI -5'Flank-7	5'-AGAAGGAAATGAGAGTCSCMTGKAGCAAAGAAAATGGGGGAA-3'	2		+				+								
PCI -5'Flank-8	5'-GAGTTGGAGAAGGAAATGGGGGAA-3'	2			+	+										
PCI -5'Flank-9	5'-AGACAGGATTGGGAGGAATGAACAGATGGGGGRRCCAAAGAAAATGGGGGAA-3'	2		+					+							
PCI -5'Flank-10	5'-TGCTCTTCCGATCTTACCTTTCCAATTATACATGTTGRAGATTCAAAGAAAATGGGGGAA	3	+	+					+							
PCI -5'Flank-11	5'-AGTAACTCAGCAGGCAATTAAAGGAAATTAGAGGCAGTCTTCAAAGAAAATGGGGGAA-	3	+					+	+							
PCI -5'Flank-12	5'-ACGATCCCATTTGGGGTTTTCTTGGCAAAGAT-3'	3	+	+		+										
PCI -5'Flank-13	5'-GTCAAAAGAGAAAATAGAATAAATGGGGGAA-3'	2				+		+								
PCI -5'Flank-14	5'-AGGAAAGGATCCATGTGCAGCAAAGAACTCGGAA-3'	2			+				+							
PCI -5'Flank-15	5'-GCTGTGGGAAGACAGGGATACTAGTGCATTGTTGGTGGAGCTATGAATCAGTACAAC-3'	2	+		+											
PCI -5'Flank-16	5'-ACCAAAACCCTTTGGGCCCTGATTGACTCAGAACAAATGTAAATAGGAATTGTTT-3'	2	+		+											
PCI -5'Flank-17	5'-TACATTATTATAAGCTGTCACTATTGCACTCATGATGCTATATATAACTATCAATCTTGG/	3	+		+	+										
PCI -5'Flank-18	5'-TTGTTCAGGACTGGATTAGACGTGTGCTCTTCGATCTRTGGGGGAA-3'	2			+				+							
PCI -5'Flank-19	5'-AAATATGAGTCAGTCCCAGGCCTTGGAAGAGCTCAAAAGGGATTTTGAGG-3'	2	+	+												
PCI -5'Flank-20	5'-TTATCCCAAAGGGAGCCTGGAAAGATATCCCAACCTTGTTAATATCAGGACTTGTCTCCA	2	+		+											
PCI -5'Flank-21	5'-CAGAAACCTTATTTGTAAAAAATTCACTTTTCTCATGGATGAACAAAGCTCTTCTGACAC	2	+	+												
PCI -5'Flank-22	5'-TGGACAGGAATTTCAGCCTTACAATTTAAAACGCAAAAATCTACCCCAGAAACAAGGAA	2	+		+											
PCI -5'Flank-23	5'-TTTGTTTATTTTAGGAAGCCACAGTAAGTCATAAAAGGGTGCAGC-3'	2	+						+							
PCI -5'Flank-24	5'-AGTAACCCTAGATCAACTTAACCCCTTGTTTTATAT-3'	4	+		+		+				+					
PCI -5'Flank-25	5'-CTCCGTAACAGTGATGATCATCTCTAGTGAGCATATATCTCCCAGTTTTGGCCTTGTCTG/	2			+				+							
PCI -5'Flank-26	5'-ATTCTTAGAATACCTGGCTTCCTTCAAGGTAAGCCCCCTTCCTATTTCTACATGAAGT-3'	3	+	+	+											
PCI -5'Flank-27	5'-CTTCAGCTGGTACGCTAGGCTTTGGAGATTTAACATGAGAAGGGTGAGTCAGTAAGGT-3	3	+					+			+					
PCI -5'Flank-28	5'-ACATGGTCTTTTCCTTTTAGGGGTTCAGATGCCATCCCCATCATACCCACTGCAACACCT/	2	+		+											
PCI -5'Flank-29	5'-TTGATCTGAGGTCCTCCTGACTTCAGGGCTGGGGCTCTATCCACTGCACCACCTGGCTGC	2	+		+											
PCI -5'Flank-30	5'-CCCCCATCCTCCTCCTCCTGGTCCTTGCCTAGTGTCAGCTTCCAAACACTCTAGTAGAGT(	2	+		+											
PCI -5'Flank-31	5'-ACATGAGAGCAGCCTGGTTTGAACTTCCTCTGGTTCTTTTCAGCTTCAGTGCAGGAA-3'	2			+		+									
PCI -5'Flank-32	5'-CTAATATGACAAAAAAAGAAAATGGGGGAA-3'	2			+				+							
' flanking sequences																
PCI -3'Flank-1	5'-TGTGAACCCTGAGCAAATCACTTAACCCCATTGCCTAGCCAAAAAAAAGCAAAACAAAA	4	+	+	+				+							
PCI -3'Flank-2	5'-GTCTGTACTCTGGACTCCATTCTCTATACTTTCACCATTTACCCCTGGGCCGGGCTCGTA(	2	+			+										
PCI -3'Flank-3	5'-AGACCATAACGAGAGGGGAGTTCAGGGGAACAAGAACAGAAGTGGGGTTCTGGTGGTG	5	+	+				+	+				+			
PCI -3'Flank-4	5'-CTATATTTCAAAAGATCTTTATTTTCACCAACAATGTATACATCCTACAGAAATCAATGT(	3	+	+	+											
PCI -3'Flank-5	5'-TCADATHGWTTCATTATACTCTGTTTCTGCGCTCAGGATTCTCTCCTTTCAACAGGCCG-3	2	+					+								
PCI -3'Flank-6	5'-CTGAGAAAGGTGGAGCTAAGCAGTATCTGTAGTTTTTCTCTTTTAGCAGTGAAAAAA-3'	2	+			+										
PCI -3'Flank-7	5'-TTTTCATTTACTCAGATTTCAGTTTCCTATTCTCGTAATCATTGTGCATATTTTTCCT-3'	2	+				+									
PCI -3'Flank-8	5'-AGACCAAACACTTGACACTTAGTAGCTGTGTGACCCTGGGCAAGTCA-3'	3		+					+	+						
PCI -3'Flank-9	5'-GAAGCAGCATTTGTGGGATCCTTTGGAAAATACTGTGAGAATAAC-3'	2	+		+											
PCI -3'Flank-10	5'-CAACCAAGCAATTAAAGCCATCAACAGCAAGSSAGAGCATGTGG-3'	4	+		+			+	+							
PCI -3'Flank-11	5'-CGAGGAGTTTGTTTGCTAAAGGATAGGAGGTGAGGGGCACA-3'	2	+	+												
PCI -3'Flank-12	5'-GTGCCTCTTATATGACAGGACTATGTAGGCACCATGTCTTTATTCCTGCTTGCAATTATAT	5	+	+				+	+			+				
PCI -3'Flank-13	5'-CTGTGAATGACCTAGCTATTCTCAGCAATGCAATGATCCAAGACCATTCTGAAGAACTCA	2	+	+												
PCI -3'Flank-14	5'-CTACATTCTGACATTTCTAGTCTTCTCCCGTTTACTTCCCTATACATACACTACCATTACC	3	+						+			+				
PCI -3'Flank-15	5'-ATAGAACATTCTCAAAGTTGGACAAACTCTCAGAAATTTGTTTGCATAAATATTCATCCG	5	+	+					+		+	+				
PCI -3'Flank-16	5'-GTACGATGAATCCAAAGCTCTTTGCTTTTTTCATCTAATCTTGTCTATCCTCCTGTTGATG	4	+	+	+							+				
PCI -3'Flank-17	5'-CTATCTTGTTTTATTTTAAAAGTCTGAAAAATACAGCAGCGTTGGAGACATAGATATGCA	5	+		+				+		+		+			

+	Light grey represents insertion sites found in two koalas
+	Middle grey represents insertion sites found in 3 kolas
+	Dark grey represents insertion sites found in four or more koalas
